# Rational therapeutic targets with biomolecular liquid-liquid phase separation regulating synergy: A pan-cancer analysis

**DOI:** 10.1371/journal.pone.0287574

**Published:** 2023-11-02

**Authors:** Si Sun, Wenwen Wang, Guoqing Li, Man Xiao, Minggang Peng, Jing Cai, Zehua Wang, Qiang Yang, Xiaoqi He

**Affiliations:** Department of Obstetrics and Gynecology, Union Hospital, Tongji Medical College, Huazhong University of Science and Technology, Wuhan, 430022, China; Xiangya Hospital Central South University, CHINA

## Abstract

Liquid-liquid phase separation (LLPS) is characterized as an ubiquitous framework for diverse biological processes including carcinogenesis and cancer progression. While targeting cancer from perspective of LLPS offers an opportunity to drug the conventionally undruggables with cancer-driving potential, the therapeutic value of cancer associated LLPS (CAL) proteins remains elusive. Here, we report the genomic landscape, prognostic relevance, immune-infiltration association, down-stream pathway alteration and small molecular responsiveness of CAL protein-coding gene signatures based on protein-coding associated mutations and transcriptional abundance in pan-cancer. Correlations of CAL protein-coding associated mutations and transcriptional abundances to overall survival and progression-free survival were observed in an array of cancers and further characterized by differential survival outcomes between patients with intrinsic disordered region (IDR) enriched and non-IDR enriched mutations in endometrial cancer. Altered signaling pathways and universal pattern of immune infiltrates on account of CAL protein-coding associated gene-set mutations involved key components of oncogenesis in various cancer types and well established therapeutic targets including MAPK signaling pathway and implied an inflamed tumor immunity that might be highly responsive to immunotherapy. LLPS inhibitor enhanced cytotoxicity of cisplatin/paclitaxel in selective cancer cell lines. These findings provide preliminary evidences for rational chemo-, targeted- and immuno-therapeutic innovation with LLPS regulating synergy.

## Introduction

Cancer cells are experts deft at taking advantages of their unusual characteristics or signatures to evolve and to stand against conventional therapy. Serial work from numerous endeavors and The Cancer Genome Atlas (TCGA) seeking for potential therapeutic opportunities have unraveled a massive repertoire of candidate cancer prognostic signatures and drug targets that gives rise to an amount of immuno- and targeted-therapeutic strategies represented by microsatellite instability as parameters for immune checkpoint inhibition therapy [1], trastuzumab targeting HER-2 in HER-2 positive cancer [2,3], Olaparib targeting PARP1 in BRCA1/BRCA2 mutant ovarian cancer and etc. [4]. Substantial underlying therapeutic targets await to be mined as the next-generation weapon to battle against cancer [5,6].

The conventional strategy for protein functional inhibition is based on screening of small molecules that can fit in the defined structures of a protein such as the classic lock-and-key interactions. However, instead of being at the lock-and-key interaction area, many promising target sequences are located in intrinsically disordered regions (IDRs) of cancer associated proteins making them hard to be drugged. IDRs are the structural basis that confers a protein multivalency to drive liquid-liquid phase separation (LLPS), a reversible process of condensation of biomolecules from a dilute phase into a condensed phase de-mixing themselves from the bulk environment in vitro and in vivo [7]. Looming evidences supporting the intimate role of biomolecular LLPS in cancer evolution and progression provide opportunities for drugging the conventionally undruggable targets from a LLPS perspective [8–10]. However, clinical relevance of cancer associated LLPS targets remain elusive.

Here, we aim to identify cancer associated LLPS (CAL) protein-coding genes and to evaluate corresponding clinical correlations between functional LLPS associated signatures and cancer survival outcomes, associated pathway alterations, differed immune infiltrates, potential drug responsiveness and enhancement of classical chemotherapy. Considering that in-vivo LLPS was driven by IDRs and reinforced by affluent protein abundance, analyses were mainly performed from aspects of CAL protein-coding associated mutations and transcriptional abundance.

## Materials and methods

### Ethical approval

This study was approved by Independence Ethics Committee of Union Hospital, Tongji Medical College, Huazhong University of Science and Technology (20210567). The informed consent was waived according to the protocol apporved by the ethics committee.

### Collection of proteins with LLPS characteristics and datasets studied

Basic information of proteins with LLPS characteristics were acquired from the PhaSepDB (http://db.phasep.pro/) [11]. PhaSepDB is a database of phase-separation-related proteins based on the curated information derived from published literature, in which a total of 961 LLPS entries were recorded. The pan-cancer data were obtained from cBioPortal (https://www.cbioportal.org).

### Protein-coding associated mutation data collection and related survival analysis grouping

Somatic protein-coding associated mutation data were collected from TCGA as previously described [12]. Mutations that might cause protein-coding alterations were referred to as protein-coding associated mutations including missense mutation, nonsense mutation, frame-shift insertion, frame-shift deletion, in-frame deletion, in-frame insertion and splice site mutations. Mutations occurred in non-protein-coding regions were deemed as non-protein-coding associated mutations. Protein-coding associated mutations occurred in IDRs were deemed as IDR associated mutations. Manually curated and experimentally validated data from PhaSepDB were referred to for IDR determination. For IDR mutation associated survival analysis in UCEC, patients were classified into IDR-enriched mutation group and non IDR-enriched mutation group according to the number of IDR associated mutations each patient harbored (cut-off = 1, median of No. of IDR associated mutations). For mutation associated survival analysis in UCEC, patients were classified into mutation enriched group and single mutation group according to the number of protein-coding associated mutation each patient harbored.

### Gene expression and regulatory network analysis

The expression profiling of CAL protein-coding genes between 31 types of cancer and corresponding non‐carcinoma organs was analyzed using the GEPIA2 web server (http://gepia2.cancer-pku.cn) based on TCGA and GTEx projects with cutoff threshold of adjusted P < 0.05 and fold change > 1.5 [13]. The mean normalized mRNA levels of CAL protein-coding genes were acquired and then normalized according to row Z scores. The critical transcription factors and miRNAs regulating the transcriptional and posttranscriptional level of collected LLPS protein were acquired on the NetworkAnalyst platform (https://www.networkanalyst.ca/) [12].

### Functional enrichment analysis

Gene Set Variation Analysis (GSVA) of CAL protein-coding genes was performed using GSVA package through a public pan-cancer analysis web server Gene Set Cancer Analysis (GSCA) (http://bioinfo.life.hust.edu.cn/GSCA/#/),(12) and the export results, GSVA scores, positively correlate with the expression of gene set and represent the integrated level of the expression of CAL protein-coding genes.

### Pathway analysis

Pan-cancer TCGA reverse phase protein array (RPPA) data were obtained from cBioPortal (https://www.cbioportal.org). Differential pathway components were submitted for Kyoto Encyclopedia of Genes and Genomes (KEGG) and Gene Ontology (GO) analyses, clusterProfiler package. Adjusted P < 0.05 was considered statistically significant. Correlation between pathway activity and gene/gene-set transcriptional level was performed using GSCA [12]. Briefly, samples were classified as high vs. low gene expression by median and the pathway activity score between the two groups is defined by student T test. Pathway score was calculated as the difference value of relative protein level of positive regulation components minus that of negative regulatory components. Genes were considered actively correlated with a pathway when the pathway score of high expression group > that of low expression group. FDR < 0.05 was considered statistically significant.

### Survival analysis

Kaplan-Meier analyses were carried out to compare the survival time difference using the survival package, and a log-rank test was utilized to test the efficiency, in which overall survival (OS), progression-free survival (PFS), disease-free survival (DFS), and disease-free interval (DFI) were analyzed. For continuous variables, univariate Cox regression analysis was performed to calculate the hazard ratio of each CAL protein-coding gene and displayed with forest plots.

### Immune infiltration analysis

The immune infiltration statuses of 24 immune cells were assessed in Immune Cell Abundance Identifier (ImmuCellAI) dataset [14]. The correlation between CAL protein-coding gene set protein-coding associated mutation, GSVA scores, transcriptional level, and the immune cells was evaluated based on the Pearson algorithm. P < 0.05 was considered statistically significant.

### Drug sensitivity analysis

IC50s of more than 600 small molecules in more than 1000 human tumor cell lines were acquired from Genomics of Drug Sensitivity in Cancer (GDSC) (https://www.cancerrxgene.org/) and the Cancer Therapeutics Response Portal (CTRP) (https://portals.broadinstitute.org/ctrp/) as well as corresponding cellular CAL protein-coding gene mRNA level. Pearson correlation was used to assess correlation between drug responsiveness and gene mRNA level.

### Cell culture

The human ovarian cancer cell line CAOV3 was obtained from the cell bank of the Chinese Academy of Science and cultured in complete media composed of 90% (V/V) DMEM and 10% (V/V) FBS. SKOV3, OVCAR3 and ES2 were all purchased from the American Type Culture Collection (ATCC, USA); SKOV3 and ES2 were cultured in complete media composed of 90% (V/V) DMEM and 10% (V/V) FBS, OVCAR3 was cultured in complete media composed of 80% (V/V) RPMI-1640 and 20% (V/V) FBS. OVCAR4 cell line was purchased from Bluefbio company and cultured in complete media composed of 80% (V/V) PRIM-1640 and 20% (V/V) FBS. Human cervical cancer cell lines Hela was obtained from the cell bank of the Chinese Academy of Sciences and cultured in complete media composed of 90% (V/V) MEM and 10% (V/V) FBS; CaSki, C33A and SiHa were purchased from ATCC and cultured in complete media composed of 90% (V/V) PRIM-1640 and 10% (V/V) FBS. Human non-small cell lung cancer cell line H1299, A549, breast cancer cell line MDA-MB-231 and bladder cancer cell line T24 were all obtained from ATCC and cultured in complete media composed of 90% recommended medium and 10% (V/V) FBS. All cell lines except MDA-MB-231 were cultured in a humidified incubator with 5% CO_2_ at 37°C, and the MDA-MB-231 was cultured in a humidified incubator at 37°C without CO_2_.

### Cell viability assay

The cell viability of tumor cells was examined using a 3-(4,5-dimethylthiazol-2-yl)-2,5-diphenyltetrazolium bromide (MTT) assay. Briefly, 1 x 10^4^ of cells were appended to a 96-well plate and cultured overnight. After being exposed to different gradient concentrations (6 replicates for each concentration) of cisplatin or paclitaxel for 24 hours, the cells were treated with 2% sorbitol or hexanediol, respectively, together with the chemotherapeutic drugs for another 24 hours. Then, 10 μL of 5 mg/mL MTT was added and incubated for 4 hours, followed by dissolution with DMSO. The spectrophotometric absorbance at 570 nm wavelength of the samples was measured by a microplate reader (SpectraMax, Sunnyvale, CA, USA).

### Cell cycle by flow cytometry

Tumor cells were treated with 10μM cisplatin or paclitaxel for 12 hours, respectively, followed by 2% hexanediol together with the chemotherapeutic drugs for another 12 hours. Then, about 5×10^6^ cells were collected and washed with ice-cold phosphate-buffered saline (PBS) and fixed in 5 mL 70% ice-cold ethanol overnight at 4°C. Subsequently, the cells were pelleted and stained with 500 μL PI/RNase staining solution (KeyGEN Bio TECH, KGA512) in dark for 60 minutes followed by 3 washes with PBS. Fluorescence of PI was measured at an excitation wavelength of 488 nm by flow cytometry on a MoFlo XDP flow cytometry (Beckman, USA) and about 20,000 cells were collected for analysis.

### Statistical analysis

Differential gene expression analysis was performed by GEPIA2. Pearson correlation and log rank test were performed using the R package. Student’s T test used to assess the differences between cell cycle distribution was performed using GraphPad Prism 8.0. P < 0.05 and FDR < 0.05 were considered significant when applicable.

## Results

### Identification of CAL protein-coding genes through pan-cancer analysis

To identify CAL protein-coding genes, official gene symbols of manually curated and experimentally validated LLPS proteins were obtained from PhaSepDB2.0 for differential gene expression analysis (DGE) between tumor and normal samples in TCGA and GTEx datasets across 31 types of cancer [11]. 22 over-expressed genes in cancer (2 with in vitro evidence: BIRC5, CDT1; 7 with in vivo evidence: USH1C, BLNK, RUNX2, CAV1, STIL, CDCA8, DACT1; 13 with in vivo FRAP evidence: PLK4, CBX5, G3BP1, USP42, MED1, TOPBP1, UBQLN2, CBX2, GATA3, TJP3, POU5F1, TJP1, YAP1) were identified and defined as CAL protein-coding genes since upregulated genes were more likely to be targeted (S1 Fig). The general landscape of interacting transcription factors and microRNAs as well as gene ontology of 22 CAL protein-coding genes were mapped (S2 Fig). Since initiation of protein LLPS required intact protein structure and adequate protein abundancy, we then explored the landscape of protein-coding associated mutations and transcriptional levels of the 22 CAL protein-coding genes in tumoral scenarios.

### CAL protein-coding associated mutations and cancer prognosis

**Fig 1:**
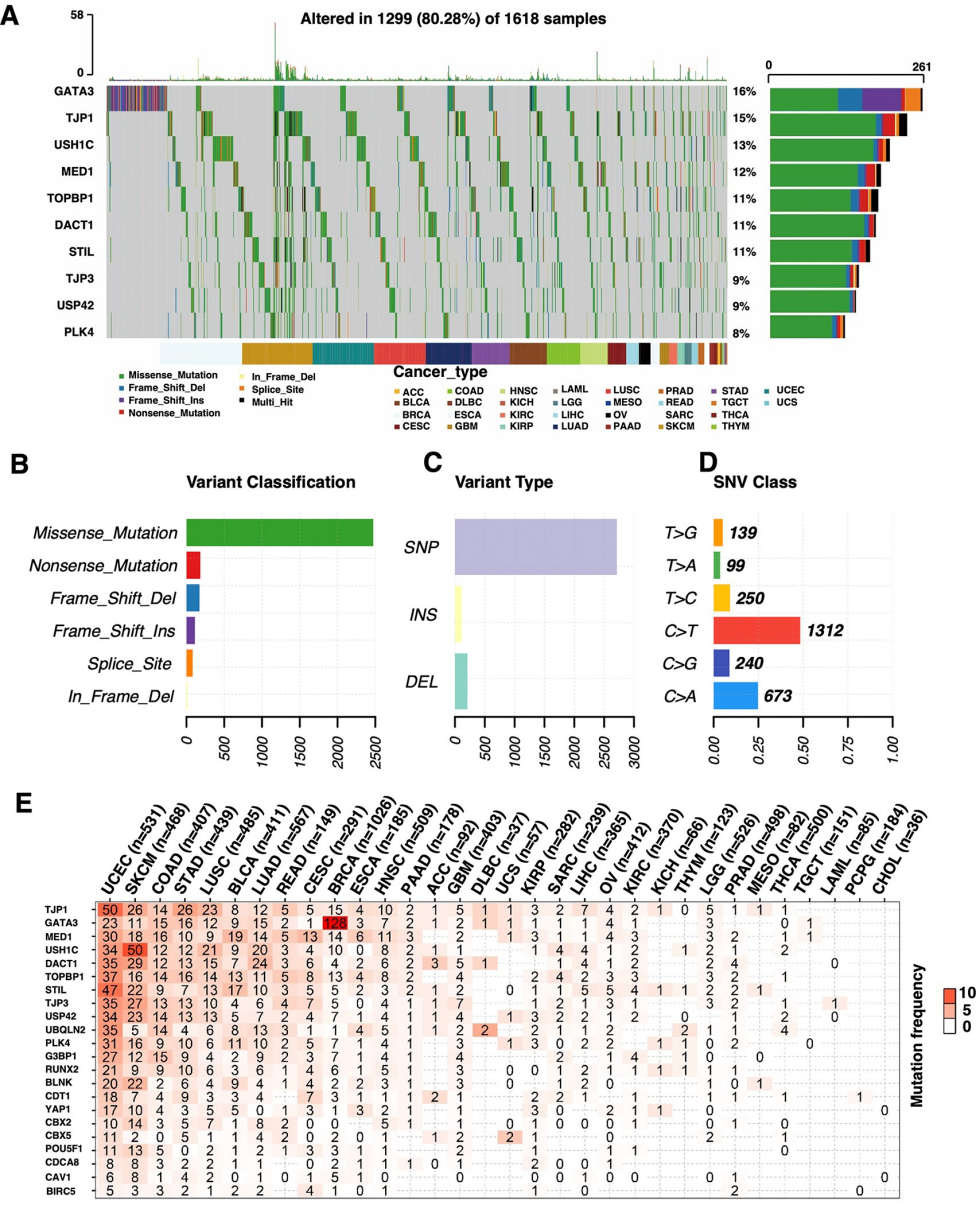
General protein-coding associated mutation status of CAL protein-coding genes in pan-cancer. (A) The waterfall plot, (B) variant classification, (C) variant type and (D) protein-coding associated mutation class of top 10 mutated CAL protein-coding genes. (E) Mutation frequencies of 22 CAL protein-coding genes in 32 types of cancers.

4280 (2639 protein-coding associated and 1641 non-protein-coding assocaited) mutations were discovered in 10234 samples of 31 types of cancers (S1 Table). Among the 1618 patients harboring at least one protein-coding associated muntation, the top 10 mutated genes included GATA3, TJP1, USH1C, MED1, TOPBP1, STIL, DACT1, TJP3, USP42, PLK4 and BIRC5, CAV1, CDCA8, POU5F1, CBX5, CBX2 and YAP1 were rarely mutated. Nearly half of protein-coding associated mutations of the Top1 mutated gene GATA3 (128/261) occurred in BRCA while the protein-coding associated mutations of 9 other genes tened to be evenly distributed across all the cancer types with a major pattern of missense mutation (Fig 1A–1D). Top 5 tumors harboring most abundant LLPS associated protein-coding gene mutations were UCEC, SKCM, COAD, STAD and LUSC, while CHOL, PCPG, LAML, TGCT, THCA and MESO seldom harbored these mutations. The mutation frequency of GATA3 in BRCA and USH1C in SKCM exceeded 10% (Fig 1E).

**Fig 2:**
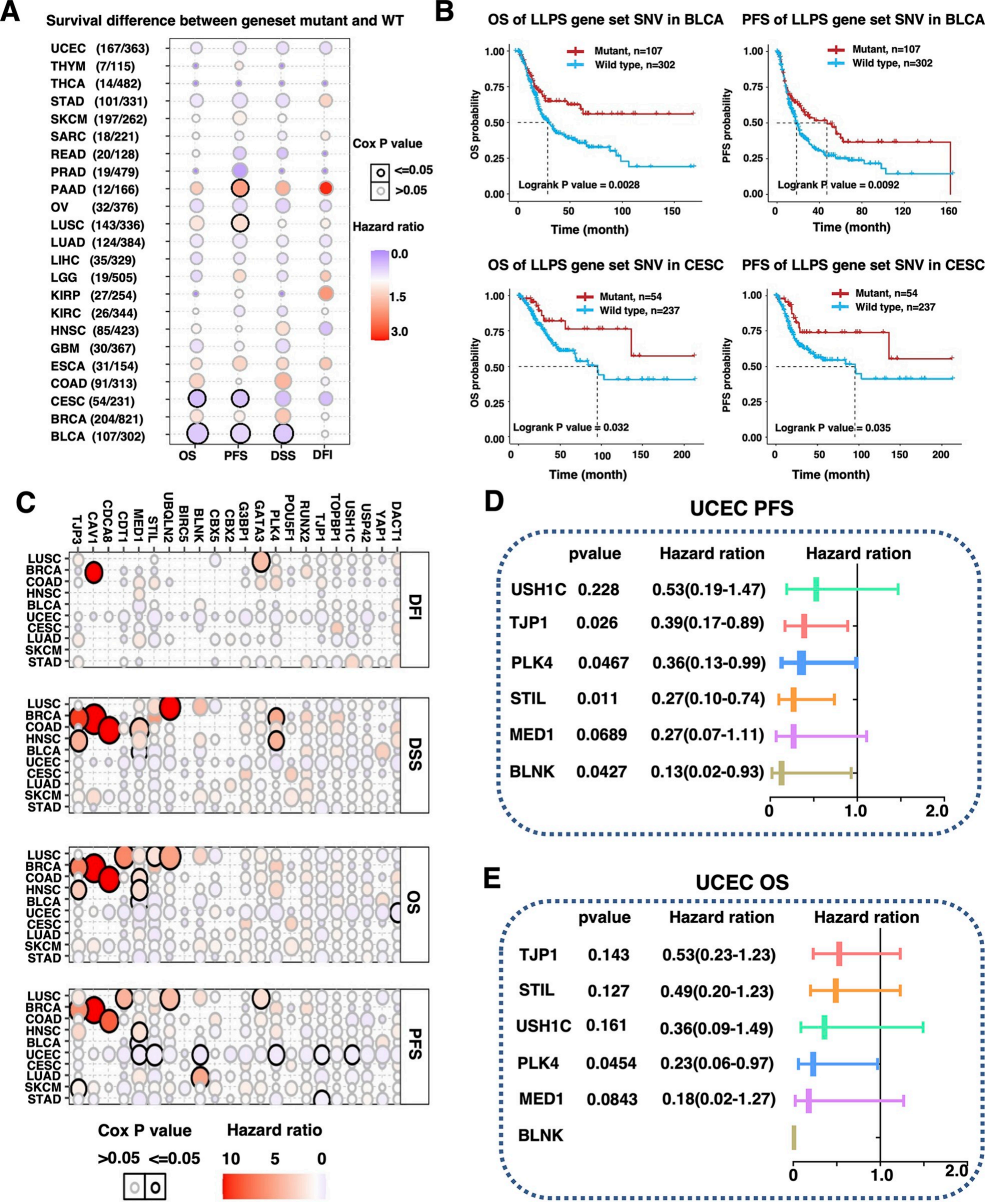
Prognostic value of CAL protein-coding associated mutations. (A) Bubble plots presenting HR of survival differences between patients harboring CAL protein-coding associated mutation and patients wild-type gene pattern in cancers with sample size≥100. (B) Kaplan-Meier plots in BLCA and CESC patients with CAL protein-coding associated gene-set mutation compared to patients with wild type gene pattern. (C) Bubble plots presenting hazard ratios of survival differences between patients with individual CAL proteins-coding associated mutation and wild type in cancers with sample size ≥ 100. (D and E) Forrest-plots presenting HR and 95%CI in UCEC patients with TJP1, USH1C, STIL, PLK4, MED1 or BLNK protein-coding associated mutation compared to patients with wild type gene pattern.

We first analyzed the correlation of gene-set protein-coding associated muntation of 22 identified CAL protein-coding genes and prognosis in 31 cancer types with 10802 cases. Overall, CAL protein-coding associated gene-set muntations demonstrated correlation tendencies with better prognosis in most cancers including GBM, LIHC, LUAD, OV, UCEC and typically in BLCA and CESC (Fig 2A and 2B). The clinical associations of CAL protein-coding associated muntation and cancer prognosis were then characterized in cancer cohorts containing at least 1 gene mutated in no less than 10 samples (Fig 2C). Notably, nearly all 22 CAL protein-coding associated muntation presented correlation tendencies with improved prognosis in UCEC, among which protein-coding associated mutations of BLNK, MED1, STIL, PLK4, TJP1 and USH1C were significantly correlated with better PFS and protein-coding associated mutation of DACT1 was correlated with better OS (Figs 2D, 2E and S3). Further analysis in UCEC suggested that patients harboring CAL protein-coding associated muntation were more likely to be classified as the pole mutation subtype (S4A and S4B Fig).

**Fig 3:**
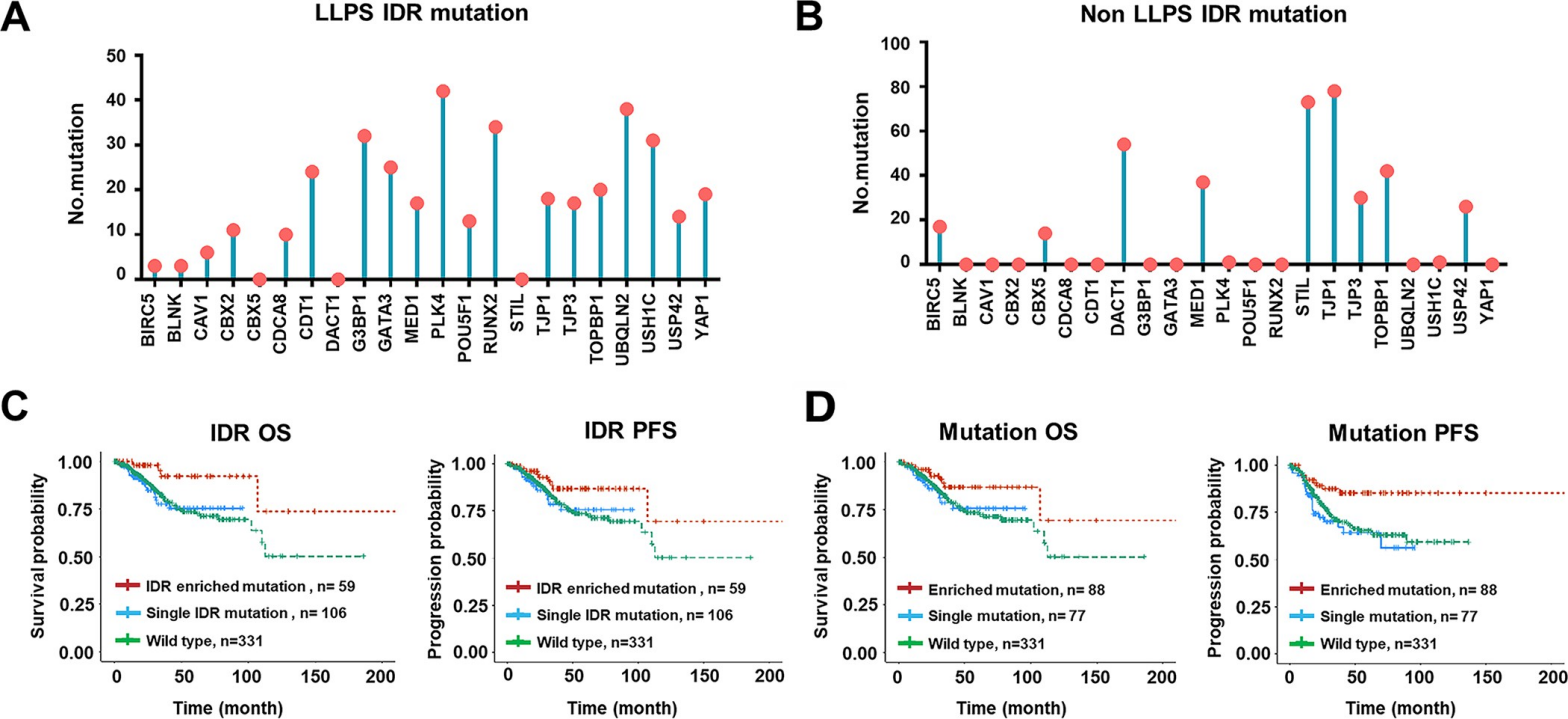
Correlation between CAL protein-coding associated mutations and improved prognosis in UCEC. (A) The general landscape of potential somatic IDR associated mutations and (B) non-IDR associated mutations were mapped in UCEC patients. Kaplan-Meier plots for OS and PFS in UCEC patients with (C) IDR enriched mutations, single IDR mutation and wild type, and in patients with (D) enriched CAL protein-coding mutations, single mutation and wild type.

The integrity of IDR was the foundation of intracellular protein LLPS. Mutation of amino acids in IDR might abrogate the ability to phase-separate of a protein [15]. To investigate the possible impact of potential IDR alteration on cancer prognosis, experimentally validated and manually curated IDR regions of CAL proteins were obtained from PhaSepDB2.0 and general landscape of IDR associated mutations of CAL protein-coding genes was mapped accordingly (Fig 3A and 3B). Patients were classified into the IDR-enriched mutation group and the single IDR mutation group. While there was no significant survival difference between patients harboring CAL protein-coding associated mutant and wild type CAL protein-coding genes (S4C and S4D Fig), patients harboring single IDR mutation had greater risk of death (HR = 3.960; 95% CI: 1.164–13.472, P = 0.028) and progression (HR = 3.578; 95% CI: 1.374–9.315, P = 0.009) compared to patients harboring IDR enriched mutations (Fig 3C). Since increased number of mutations might increase the probability of protein product dysfunction. Patients were then classified into the CAL protein-coding associated mutation enriched group and the single CAL protein-coding associated mutation group. Patients harboring single CAL protein-coding associated mutation had greater risk of progression (HR = 2.857; 95% CI: 1.350–6.048, P = 0.006) compared to patients harboring enriched CAL protein-coding gene mutations (Fig 3D). These results suggested survival predictive value of CAL protein-coding associated mutations and CAL protein-coding IDR associated mutations in UCEC.

### Transcriptional level of CAL protein-coding genes and cancer prognosis

**Fig 4:**
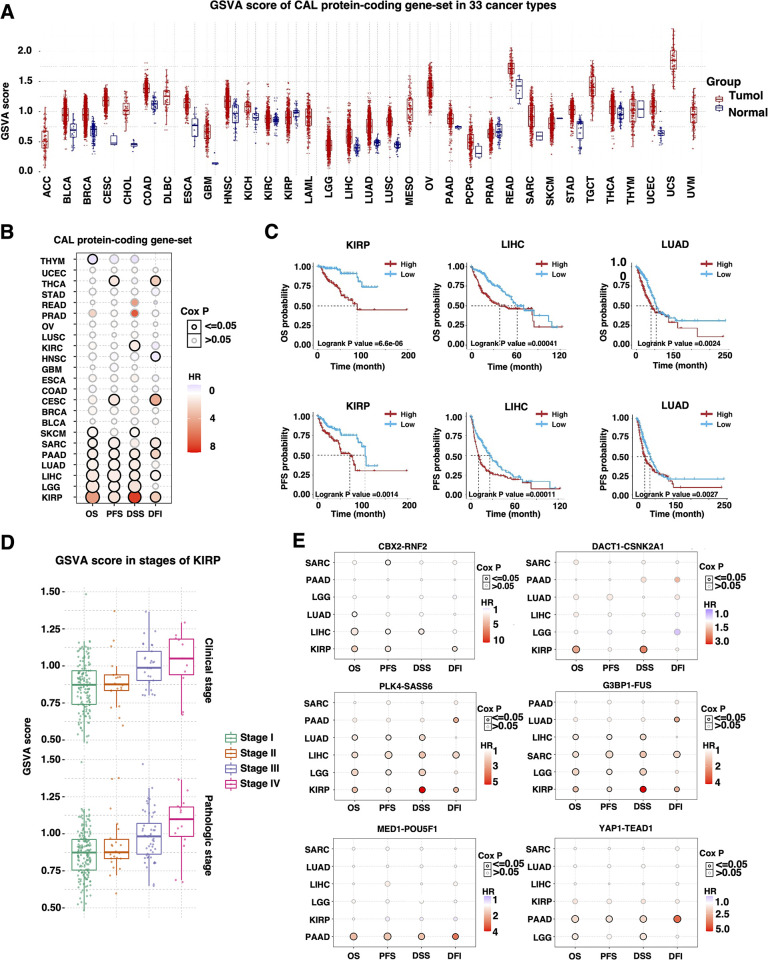
Prognostic value of CAL protein-coding gene-set at transcriptional level. (A) GSVA score of CAL protein-coding genes across 31 types of cancers. (**B**) Bubble plots representing the HRs of survival differences between high vs low CAL protein-coding gene-set GSVA in cancer with cohorts containing at least 100 patients. (**C**) Kaplan-Meier plots in KIRP, LIHC and LUAD patients with high vs low CAL protein-coding gene GSVA score. (**D**) Box plots demonstrating the distribution of CAL protein-coding gene-set GSVA among different clinical and pathologic stages in KIRP (**E**) Bubble plots representing the HRs of survival differences in KIRP, LIHC, LUAD, LGG, PAAD and SARC between patients with high and low CBX2/RNF2, G3BP1/FUS, DACT1/CSNK2A1, PLK4/SASS6, MED1/POU5F1 and YAP1/TEAD1 GSVA scores.

To evaluate the correlation between transcriptional level of CAL protein-coding genes and pan-cancer prognosis, CAL protein-coding gene-set GSVA scores were acquired as previously described (Fig 4A) [16]. Generally, high GSVA score of CAL protein-coding gene-set was associated with increased risk of unfavorable prognosis in KIRP, LGG, LIHC, LUAD, PAAD SARC, SKCM, CESC, KIRC and THCA with the most evident significance in KIRP (Fig 4B and 4C). Further in KIRP, high CAL protein-coding gene-set GSVA correlated with increased clinical and pathologic stages (Fig 4D). BIRC5, STIL, CDT1, CDCA8, G3BP1 and PLK4 shared similar prognostic pattern in LCC, KIRP, LIHC and KIRC (S5 Fig). Among the 22 identified CAL protein-coding gene products, CBX2, DACT1, G3BP1, MED1, PLK4 and YAP1 were self-drive LLPS proteins and their LLPS clients RNF2, CSNK2A1, FUS, POU5F1, SASS6 and TEAD1 were identified from the PhaSepDB2.0 database to form six LLPS protein-coding gene pairs for subsequent GSVA & survival analysis in cancers in which CAL protein-coding gene-set GSVA was of prognostic value for both OS and PFS. The correlation between enrichment of four out of six LLPS protein-coding gene pairs and poor prognosis in KIRP suggested extensive involvement of CAL protein-coding genes in cancer progression and malignant biological behaviors in KIRP (Fig 4E).

### Immune infiltration of cancers with different CAL protein-coding associated mutations and transcriptional status

**Fig 5:**
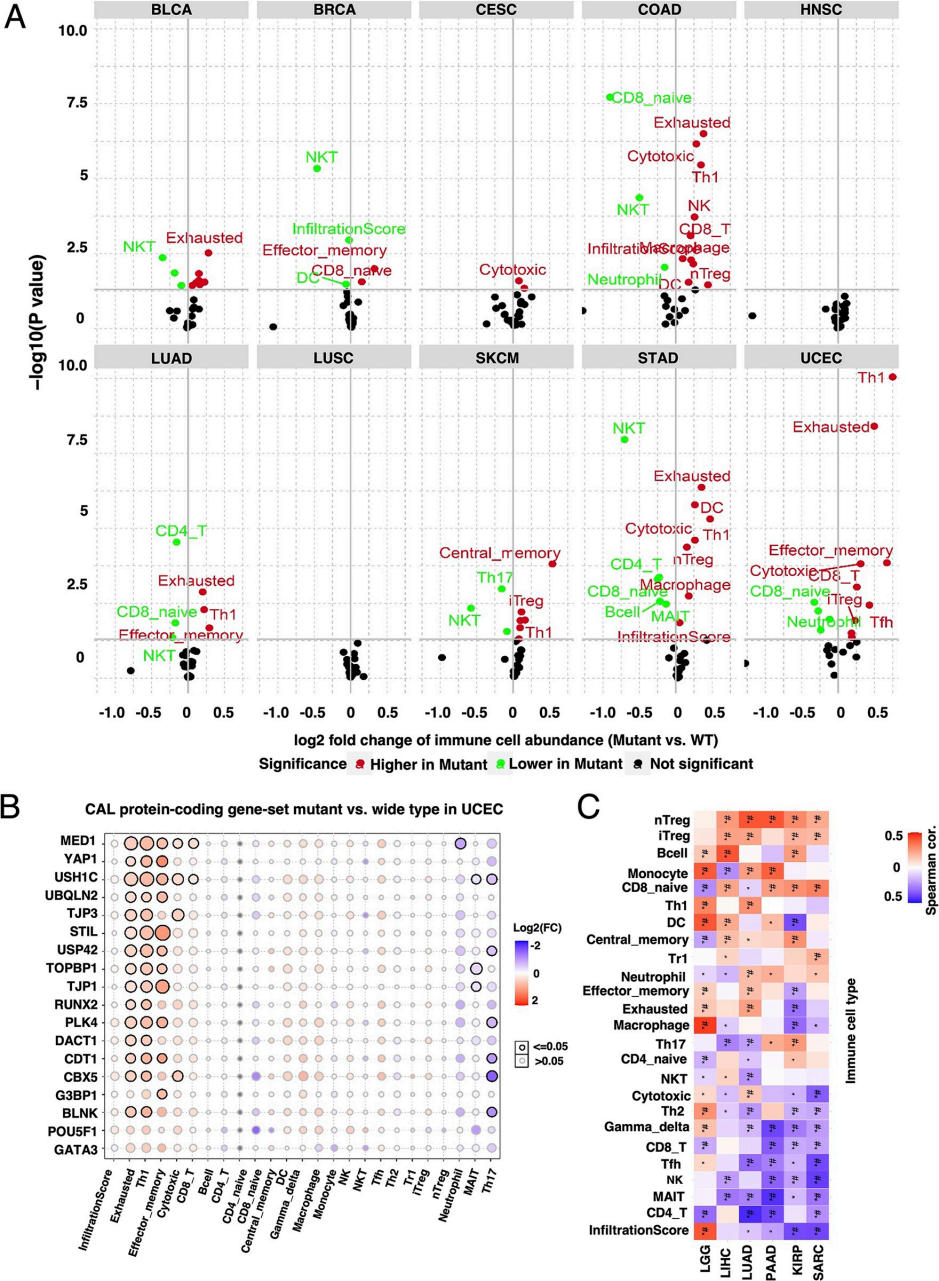
Correlation between CAL protein-coding associated mutations and immune infiltration pattern. (A) Volcano plots summarizing the difference of immune infiltration between patients harboring CAL protein-coding associated gene-set mutations and patients with wild type CAL protein-coding genes. (B) Bubble plots representing the immune infiltrates between mutant and wild type of individual CAL protein-coding genes in UCEC. (C) Heatmap representing the spearman correlation between GSVA score of CAL protein-coding gene set and immune infiltrates.

In order to test whether CAL protein-coding gene signatures could be efficient markers to predict responsiveness of immunotherapy, correlation between CAL protein-coding associated mutations, mRNA level and immune infiltration were evaluated. CAL protein-coding associated gene-set mutations was correlated with increased infiltration of cancerous cell killer Th1, cytotoxic T, effector memory T and exhausted T cells in UCEC, STAD and COAD with high MSI status (Fig 5A). In UCEC, the same infiltration pattern was observed among samples harboring mutations of genes regulating DNA damage repair and cell cycle but not in patients with mutations of classical cancer hallmarks such as PTEN, MYC, KRAS, BAX and ect. (Figs 5B, S6 and S7). In cancers in which CAL protein-coding gene GSVA were of prognostic value, the diversity of immune infiltration statuses posed great challenges to finding any specific signatures (Fig 5C).

### CAL protein-coding gene associated molecular pathways in pan-cancer analysis

**Fig 6:**
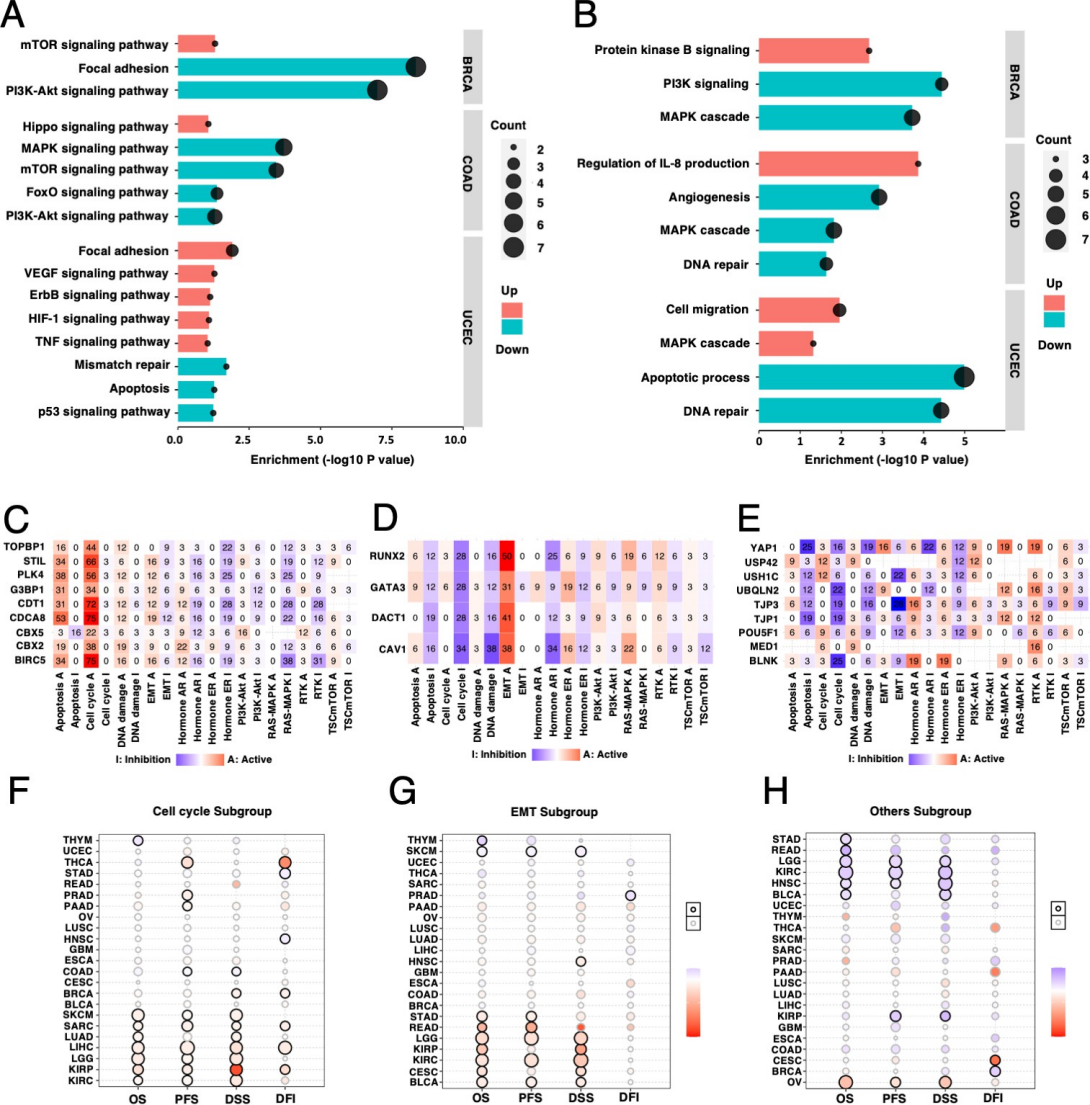
CAL protein-coding gene associated pathway alteration in pan-cancer. (A) Enriched KEGG pathway and (B) GO biological processes in patients harboring CAL protein-coding associated gene-set mutations in UCEC, COAD and BRCA. CAL protein-coding genes classified in to pathway score cluster A (C), B (D) and C (E) by the percentages of the correlation between single CAL protein-coding gene transcriptional level and 10 classical cancer associated pathway activities across 31 types of cancer. (F-H) Bubble plots representing the HRs of survival differences between high vs low pathway score cluster A, B and C in cancer cohorts containing at least 100 patients.

Since CAL protein-coding associated gene-set mutations and transcriptional level presented prognostic of cancer, identifying related molecular pathways might be of significant referential value for targeted therapy. Adequate data were collected in UCEC, COAD and BRCA for subsequent gene-set mutation analyses. CAL protein-coding associated gene-set mutations correlated with increased level of focal adhesion components regulating cell migration, MAPK cascade and TNF signaling pathways, and with decreased DNA repair and corresponding apoptotic process in UCEC; with increased hippo signaling pathway and regulation of IL-8 production, and with decreased MAPK signaling pathway and key components regulating angiogenesis in COAD; and with increased protein kinase B signaling and decreased MAPK/PI3K-Akt cascade in BRCA (Fig 6A and 6B).

The correlation between transcriptional level of CAL protein-coding gene-set and 10 vital cancer associated pathways were investigated as previously described [12,17]. Pathway score classification highlighted three CAL protein-coding gene subgroups across 31 types of cancer. Pathway score cluster A (BIRC5, CBX2, CBX5, CDCA8, CDT1, G3BP1, PLK4, STIL and TOPBP1) demonstrated prominent correlation with cell cycle activation and apoptosis regulation (Figs 6C and S8A), pathway score cluster B (CAV1, DACT1, GATA3 and RUNX2) exhibited significant correlation with EMT promotion and cell cycle inhibition (Figs 6D and S8B), and pathway score cluster C (BLNK, MED1, POU5F1, TJP1, TJP3, UBQLN2, USH1C, USP42 and YAP1) was characterized by RTK/MAPK activation and EMT/cell cycle inhibition (Figs 6E and S8C). Each pathway score cluster revealed specific prognostic value in certain types of cancer (Fig 6H–6J).

### Drug sensitivity in pan-cancer cell lines based on CAL protein-coding gene transcriptional level

**Fig 7:**
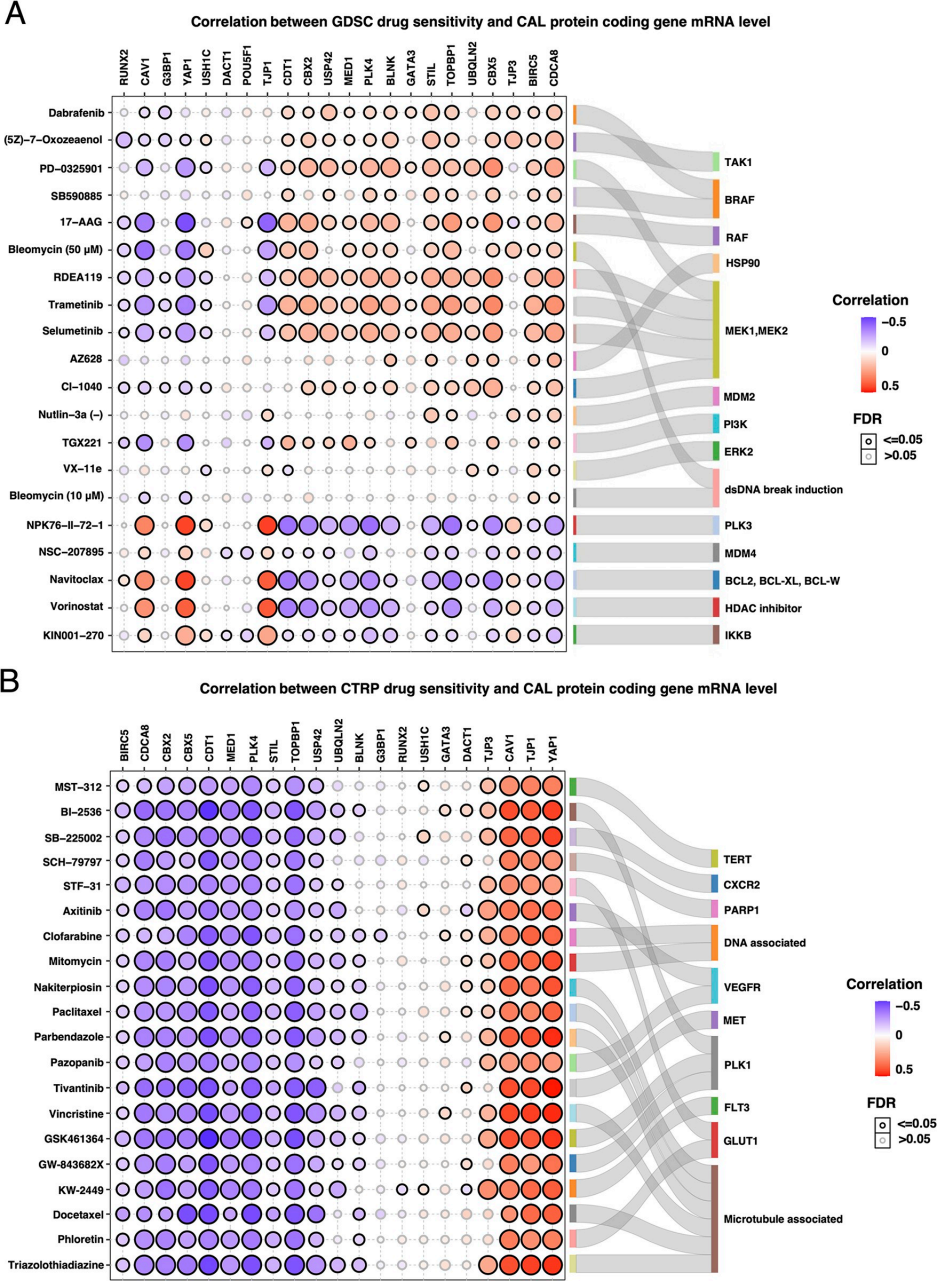
Correlation between GDSC and CTRP drug sensitivity and CAL protein-coding gene transcriptional level. (A) and (B) Bubble plots represent the top 30 correlated drugs to CAL protein-coding gene expression (left) and sanky plots represent the targets of the drugs (right).

To further evaluate the responsiveness of cells with different CAL protein-coding genic transcriptional background to existing small molecular inhibitors and chemical agents, drug responsiveness of 265 small molecules in 860 cancer cell lines from GDSC and 481 small molecules in 1001 cell lines from CTRP were analyzed on basis of CAL protein-coding gene mRNA level [18,19]. The biological function of specific drug targets were annotated through KEGG and BP of GO. CAL protein-coding genes were classified into three clusters based on cellular drug responsiveness. High transcriptional level of genes from drug response cluster A (BIRC5, BLNK, CBX2, CBX5, CDCA8, CDT1, MED1, PLK4, STIL, TOPBP1, UBQLN2 and USP42) correlated with increased IC50 to MAPK/ErbB/FoxO signaling pathway inhibitors and decreased IC50 to inhibitors of potent anti-apoptotic pathway components, DNA or microtubule toxic agents. The correlations between transcriptional level of genes from drug response cluster B (CAV1, TJP1, TJP3 and YAP1) and cellular IC50s to aforementioned drugs were completely opposite to cluster A (Figs 7A, 7B and S9A-S9D). Transcriptional level of genes in drug response cluster C (DACT1, GATA3, G3BP1, POU5F1, RUNX2 and USH1C) did not exhibit any prominent correlation with sensitization or resistance to drugs tested. Since only 1 of 22 CAL protein-coding associated mutation was detected in 5 of 900 cell lines from GDSC, we failed to analyze the correlation between CAL protein-coding associated mutations and drug responsiveness.

### Enhancement of cisplatin/Paclitaxel cytotoxicity by phase separation inhibitor in certain cancer cell lines

**Fig 8:**
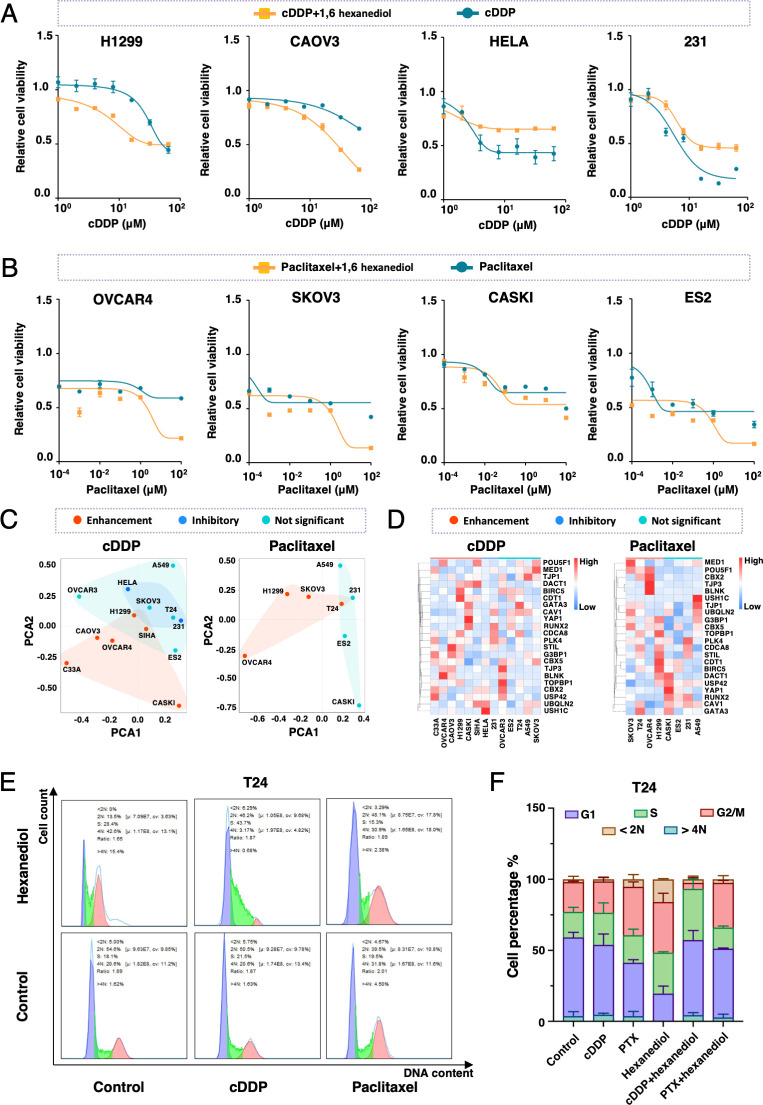
Pervasive phase separation inhibitor alters cellular sensitivity to chemotherapeutic drugs and cell cycle. (A) Cellular relative viability of H1299, CAOV3, HeLa and 231 measured by MTT exposed to cDDP alone vs. cDDP plus 2% hexanediol. (B) Cellular relative viability of OVRCA4, SKOV3, CASKI and ES2 measured by MTT exposed to paclitaxel alone vs. paclitaxel plus 2% hexanediol. (C) PCA analysis based on cellular transcriptional level of 22 CAL protein-coding genes and cellular drug responsiveness. (D) Heat map demonstrating relative transcriptional level of 22 CAL protein-coding genes across cells with different cellular drug responsiveness. (E) Representative FACS images of cell cycle distribution of T24 treated with cDDP (10 μM), paclitaxel (10 μM) for 24 hours or treated with cDDP (10 μM), paclitaxel (10 μM) for 12 hours followed by incubation with 2% hexanediol for another 12 hours. (F) The quantification of cell cycle distribution of T24. Mean ± SD of three independent experiments.

To validate whether phase separation contributed to chemo-sensitization, non-specific phase separation inhibitor 1,6-hexanediol was administered in combination with cisplatin or paclitaxel to 14 cell lines (Figs 8, S10 and S11). Inhibition of general molecular phase separation by hexanediol increased cytotoxicity of cisplatin and paclitaxel in SiHa, C33A, Caski, CAOV3, OVCAR4, H1299, and in OVCAR4, H1299, SKOV3, T24 respectively, but weakened cisplatin cytotoxicity in HeLa and 231 (Fig 8A and 8B). In order to see if there were specific cellular transcriptional patterns of the 22 CAL protein-coding genes among the cisplatin/paclitaxel sensitized or desensitized cells by 1,6-hexanediol, cell lines were sorted into diverse groups according to their reaction to 1,6-hexanediol and mapped based on transcriptional level of 22 CAL protein-coding genes (Fig 8C). Cells sensitized by 1,6-hexanediol to cisplatin and paclitaxel were characterized by increased G3BP1, STIL, PLK4, CBX5, BLNK, CBX2 and TOPBP1 mRNA levels (Fig 8D). Since these genes were formerly assessed as cell cycle associated and the overall effect of 22 CAL protein-coding genes were intimately involved in cell cycle regulation across most types of cancer, cellular cell cycle statuses were evaluated in response to 1,6-hexanediol alone or in combination with cisplatin or paclitaxel. The percentages of G2/M cells increased in T24 and C33A and decreased in Caski in response to hexanediol suggesting that phase separation inhibition might cause S or G2/M blocking or cell cycle initiation blocking in different cell types (Figs 8E, 8F and S12). Hexanediol caused S phase blocking in combination with cisplatin in T24 and C33A and promoted S to G2/M transition in Caski suggested that phase separation inhibition highly likely interfered DNA replication and cell cycle checkpoint pathways. In T24, the G2/M blocking by combination of hexanediol and paclitaxel was comparable to the effect by single agent. However in C33A, G2/M blocking by drug combination significantly exceeded the blocking caused by single agent of either hexanediol or paclitaxel. Our results demonstrated that non-specific LLPS inhibitor 1,6-hexanediol might strengthen or weaken the cytotoxicity of cisplatin and paclitaxel in vitro and suggested that cellular biomolecular LLPS was involved in chemotherapy reaction.

## Discussion

The landscapes of tumor evolution, coding and non-coding somatic cancer drivers in primary and metastatic malignancies, genomic basis for RNA alterations, circular ecDNA and cancer proteomic atlas were mapped and constantly updated due to endeavors in scales of whole-genome, transcriptional, single-cell sequencing, proteomic and all sorts of high throughput technologies. Comparing to conventional well established cancer signatures, biomolecular LLPS is a completely new member to the family of mechanisms of cancer. The clinical landscape of biomolecules with in vivo LLPS property remains elusive and is worth exploring since the basic multi-omics conditions that may contribute to in vivo biomolecular LLPS including gene mutations involving protein structural alterations and transcriptional level impacting protein abundance are affluent and mature. The newly deciphered mechanism and function of cancer associated biomolecular LLPS in return can help interpreting precedential unexplained biological and clinical events.(15) Here we describe the clinical implication of a group of CAL protein-coding genes and their loci products as well as testing the feasibility of improving cancer treatment through interfering biomolecular LLPS.

Among all clusters of cancer associated events, biomolecules with intracellular LLPS characteristics drew our attention during exploration for novel cancer treatment strategy for the following reasons: 1) as fundamental biochemical process, LLPS lied in the intersection of integration of genomic landscapes and down-stream effects [20,21], 2) biomolecules with IDR and self-drive LLPS propensity tended to be the key regulators of crucial cancer associated processes such as sustained proliferation, replication immortality, genome instability, angiogenesis, evading cell death and growth suppression as well as invasion and metastasis [22,23], 3) exploring promotion of cancer development from perspective of dynamic interaction of self-drive LLPS biomolecules and its clients added evidence to existing tumor landscapes from a novel dimension [15], 4) the molecular function of cancer associated molecules with IDRs lacking classic lock-and-key dots that were previously deemed undruggable might be blocked through LLPS disturbance [24].

In this study, we obtained a group of CAL protein-coding genes with varied “cancer popularity”. Some of the CAL protein-coding genes had already been studied profoundly in cancer. *YAP1*, encoding the key component of Hippo pathway, was activated prevalent in various cancers including oral squamous cell carcinoma, cervical squamous cell carcinoma and hepatocellular carcinoma and coupled with extensive oncogenic characteristics and processes such as *KRAS* mutation, cancer stem cell maintaining, EMT promotion, tumor immune evasion [25]. *BIRC5*, encoding the famous inhibitor-of-apoptosis protein survivin, was the central hub gene of complex molecular networks including chromosomal passenger complex, spindle formation and checkpoint control, microtubule associated spindle formation, anti-apoptosis and mitochondrial dynamics [26]. *GATA3* was frequently mutated in a variety of cancer including breast cancer or T cell leukemia, regulated luminal cell and T cell fate, and was referred to as representative molecular portraits together with *TP53*, *ESR1*, *KMT2C*, *NCOR1*, *AKT1* and ect. [27,28]. *MED1*, encoding crucial transcription regulating transcription factor, was recently reported to form super-enhancer through LLPS and coupled with cisplatin partitioning in nuclear condensates [29,30]. Some were rising stars of cancer research. *G3BP1*, encoding the core components of stress granules, promoted DNA binding and activation of cGAS pathway, might unfold an unknown vision between microbe and cancer. The cancer associated biological function of some were known little about, such as CBX2, CBX5, CDT1, TOPBP1 and RUNX2, but whose implication in cancer worth further exploration based on emerging evidences. Several of CAL protein-coding genes had long been potential targets for targeted therapy, such as YAP1 and survivin, but deemed as “undruggable” or without optimal targeted agents [31]. The correlation between CAL protein-coding genes and specific cancer survival, cancer hallmark pathway scores in pan-cancer cohorts, and cellular drug responsiveness based on GDSC and GTRP provided rationale for targeted therapy modification through LLPS interfering perspective.

Although in-vivo LLPS was increasingly acknowledged as a precedent hidden driver for oncogenesis and cancer progression, in-depth mechanisms at molecular basis were mainly unraveled in liver, lung and hematologic tumors. Evidence regarding how LLPS was involved in urogenital malignancies was scarce. In this study, CAL signatures with prognostic significance were found in urogenital malignancies including BLCA, CESC, KIRP and UCEC with several measurements of significance in UCEC. Better prognosis for patients harboring CAL protein-coding mutations compared to wild type in CESC and BLCA implicated possible participation of biomolecular LLPS in malignant biological behavior. The distinct survival outcomes between patients from the IDR-enriched mutation group and the single IDR mutation group in UCEC not only highlighted the necessity of IDR’s integrity in bio-condensates’ function but also implied the possibility of improving cancer prognosis through disturbance of IDR’s function. These results demonstrated the involvement of biomolecular LLPS in progression of urogenital malignancies and proved the worthy of future exploration of in-vivo LLPS in relevant fields.

When evaluating the prognostic value of CAL protein-coding genic transcriptional abundancies, KIRP stood out among all the other cancers. Poor prognosis of KIRP had intimate correlation with high CBX2, BLNK, PLK4, STIL and BIRC5 at single-genic transcriptional level. According to CTRP drug sensitivity analysis, high level of CBX2, BLNK, PLK4, STIL and BIRC5 were associated with increased sensitivity to inhibitors of VEGF and MET pathways, which happened to be two crucial driver pathways of renal cell carcinoma [32,33]. While surgery remained first-line treatment for early-stage renal cell carcinoma, the advent of targeted therapy improved overall prognosis of patients with advanced stages. For KIRC, the most common subtype of renal cell carcinoma, bulky studies on molecular background and biology had generated extensive evidence about efficacy of molecular targeted agents. However for KIRP, even as the most frequent sub-type of non-KIRC, it was still a rare and heterogenous malignancy that lacked high-level evidence to determine appropriate late-stage management. A recent randomized trial demonstrated improving PFS and OS in MET driven KIRP patients received MET inhibitor cabozantinib over sunitinib [34]. Even though cabozantinib had advantage over other MET inhibitors on patients harboring D1228N, M1250T or H1094/L145 mutations, it still had its limitation. Therefore mining MET relevant CAL proteins with precedent evidence might uncover novel therapeutic targets with currently unknown mechanism.

LLPS-driven biomolecular condensates were intimately involved in transcriptional regulation, DNA repair, stress response and signal transduction while 1,6-hexanediol is one of the most commonly used non-specific LLPS inhibitors during functional research of LLPS at cellular level. Recent evidence suggested that 1,6-hexanediol inhibited angiogenesis by suppression of cyclin-A1 dependent endothelial function [35]. In this study, we found that the reaction of different types of cancer cells to chemotherapy agents cisplatin and paclitaxel might change when combined with 1,6-hexanediol, which suggested that cellular biomolecular LLPS was involved in chemotherapy reaction. However, non-specific LLPS inhibitors did not target any specific targets and might come along with side effects, therefore investigating specific LLPS events would provide new possibility in targeted therapeutic design. BRD4 inhibition by JQ1 was a typical example of interfering LLPS of MED1/BRD4 at super enhancer that disrupted down-stream signaling and inhibited tumor cell proliferation. The anti-tumor effect of JQ1 was validated in an array of cancers [36]. A study by Xie et al reported their identification of ET516 as an AR LLPS inhibitor which disrupted AR transcription and inhibited the growth of castration-resistant prostate cancer [37]. Despite relatively rare successful aforementioned examples, efforts to identify target-specific phase separation inhibitors were still facing huge challenges: LLPS was a complex and dynamic processes that involved multivalent interactions between proteins and nucleic acids; in addition, the flexibility of the IDR region that drives proteomic LLPS makes screening of specific LLPS inhibitors even more resource consuming. Therefore, despite the emergence of clinical translational research for appliance of biomolecular LLPS mechanism in immuno- and targeted-therapy, the clinical application of LLPS specific inhibitor would need much more time. Besides, work devoted to unravel the underlying pattern of in vivo LLPS in conventional chemotherapy was still limited. Though the underlying mechanism exploration and exploiting was still full of thorns, the selective partition of small molecular cancer therapeutics including cisplatin by nucleic LLPS condensates flared the long-standing expedition of overcoming conventional chemotherapy resistance and improvised a visionary possibility [30].

Immunotherapy represented by immune checkpoint inhibitors, engineered chimeric antigen receptor T cells, nonspecific immunomodulator and tumor vaccine had shown promising clinical value in a variety of cancers during the last decade. It was highly praised of long response duration and minimum adverse effects but the therapeutic responses were only constrained to a fraction of patients with certain immune profile [38]. Massive studies dived into the complexity of dynamic equilibrium between tumor immunity, tumor composition and tumor micro-environment generating immune profiles, hallmark mutational signatures and expression signatures including cancer-immune set point, immunophenoscore, the TCGA immune TME subtypes and the visual global tumor portraits to predict immunotherapy response [39,40]. Here, CAL protein-coding gene expression pattern were far from optimal markers for immunotherapy response prediction. Nevertheless, the universal immune infiltration pattern representing an active immuno-responsive profile and the co-occurrences of CAL protein-coding associated mutations and POLE mutation in UCEC provided evidence for rational therapeutic design.

Unlike other static cancer associated signatures, transient dynamic of in vivo biomolecular LLPS posed great challenge to its clinical evaluation. Real-time LLPS processes in multiple cancer samples were hard to capture without support of specialized confocal microscopy and fluorescent tracing. Therefore we used static parameters including CAL protein-coding mutations and transcriptional abundance as substitutes for visualization of LLPS when evaluating clinical associations of biomolecular LLPS. However, not only the driver but also the adapter are required for functional consequence of in-vivo biomolecular LLPS. This “driver-adapter” effect could only be studied through complex IDR mutations that deprived target protein the ability to phase separate or through artificial LLPS enhancing system such as optodroplet or CORELET system and hard to be achieved through data mining [41].

Our study mainly focused on CAL protein-coding genes and their protein products on basis of existing validated repertoire. The complement of cancer-LLPS panorama of an ultimate multi-omics dimension still required relevant genomics, transcriptomics, proteomics and metabolomics data on non-coding genes, RNAs and chromosomes fueled by bulk exquisite fundamental research validation. Since LLPS ubiquitously took place in 4D environment, the boosting cutting-edge technology spatial omics might be the cornerstone to new breakthroughs of LLPS evaluation for cancer molecular classification, prognosis prediction and personalized therapy implication in clinical setting when evolved into single-cell resolution [42]. Novel molecular subtype in cancer from perspective of functional LLPS might be composed for LLPS based personalized targeted therapy.

## Conclusion

The CAL signatures identified were intimately correlated with cancer prognosis especially urogenital malignancies. CAL protein-coding associated mutations correlated with better OS and PFS in BLCA and CESC patients, and increased infiltration of Th1, cytotoxic T, effector memory T and exhausted T cells in UCEC, STAD and COAD. CAL protein-coding gene IDR mutation associated with prolonged OS and PFS in UCEC patients. High CAL protein-coding gene transcriptional abundance correlated with significant poor prognosis in KIRP and altered sensitivity to specific targeted therapy. Phase separation inhibitor enhanced cisplatin and paclitaxel cytotoxicity in cancer cells. CAL signatures might be potential therapeutic targets with LLPS regulating synergy.

## Supporting information

S1 Fig Differentially expressed LLPS protein-coding genes in pan-cancer. Gene expression level for LLPS protein-coding genes with in vitro, in vivo and in vivo FRAP evidence in 31 types of cancers. The Log2FC of each LLPS protein_coding gene of cancer vs normal. The top 25 most differentially expressed genes were presented.
(TIF)

S2 Fig General landscape of CAL protein-coding genes. The interaction net-work of CAL protein-coding gene associated transcription factors (A) and micro-RNAs (B). (C) Gene-ontology of CAL protein-coding genes.
(TIF)

S3 Fig Correlation between specific CAL protein-coding gene SNV and cancer prognosis in UCEC. Kaplan-Meier plots for OS and PFS in BLCA and CESC patients with TJP1, USH1C, STIL, PLK4, MED1 or BLNK SNV compared to patients with wild type gene pattern. OS, overall survival; PFS, progression-free survival.
(TIF)

S4 Fig Correlation between CAL protein-coding gene SNV and clinical characteristics of UCEC. (A) Distribution of UCEC patients with CAL proein-coding gene SNVs across different molecular subtypes. Chi-square P<0.001. (B) The correlation heatmap representing mutation co-occurrence and exclusion analyses for CAL protein-coding genes and POLE. ***p<0.001, **p<0.01, *p<0.05. (C) and (D) Kaplan-Meier plots for OS and PFS in UCEC patients with CAL protein coding gene SNV vs. CAL protein coding gene wild type.
(TIF)

S5 Fig Prognostic value of individual CAL protein-coding gene transcriptional level. Bubble plots representing the HRs of survival differences of OS, DFS, DSS and DFI in cancer with cohorts containing at least 100 patients.
(TIF)

S6 Fig Correlation between DNA damage/cell cycle associated genic SNV and immune infiltration pattern in UCEC. (TIF)

S7 Fig Correlation between classic UCEC associated genic SNV and immune infiltration pattern. (TIF)

S8 Fig Correlation between GSVA score of pathway score cluster A, B, C and 10 classic cancer pathway activities. (TIF)

S9 Fig The pathways and biological functions of drug targets with increased IC50s from CAL protein-coding gene drug response clusters. IC50s of inhibitors regarding targets and pathways in (A, C) and (B, D) were increased in cells with increased CAL protein-coding gene drug response cluster A and cluster B mRNA levels.
(TIF)

S10 Fig The effect of pervasive phase separation inhibitor hexanediol to cellular responsiveness to cDDP in different cell lines. Mean ± SD of three independent experiments.
(TIF)

S11 Fig The effect of pervasive phase separation inhibitor hexanediol to cellular responsiveness to paclitaxel in different cell lines. (TIF)

S12 Fig The effect of pervasive phase separation inhibitor hexanediol to cell cycle change in cells treated with cDDP and paclitaxel. (A and C) Representative FACS images of cell cycle distribution of C33A and Caski treated with cDDP (10μM), paclitaxel (10μM) for 24 hours or treated with cDDP (10μM), paclitaxel (10μM) for 24 hours followed by incubation with hexanediol (2%) for another 12 hours. (B and D) The quantification of cell cycle distribution of C33A and Caski. Mean ± SD of three independent experiments. Statistical P please refer to S2 Table.
(TIF)

S1 Table Summary of individual CAL protein coding genes in pan-cancer. (XLSX)

S2 Table Cell cycle statistics. (XLSX)
