# Supplementary figures and images for: Rational therapeutic targets with biomolecular liquid-liquid phase separation regulating synergy: A pan-cancer analysis
Source: PLoS One. 2023 Nov 2;18(11):e0287574. doi: 10.1371/journal.pone.0287574 (PMC10621828; doi:10.1371/journal.pone.0287574)

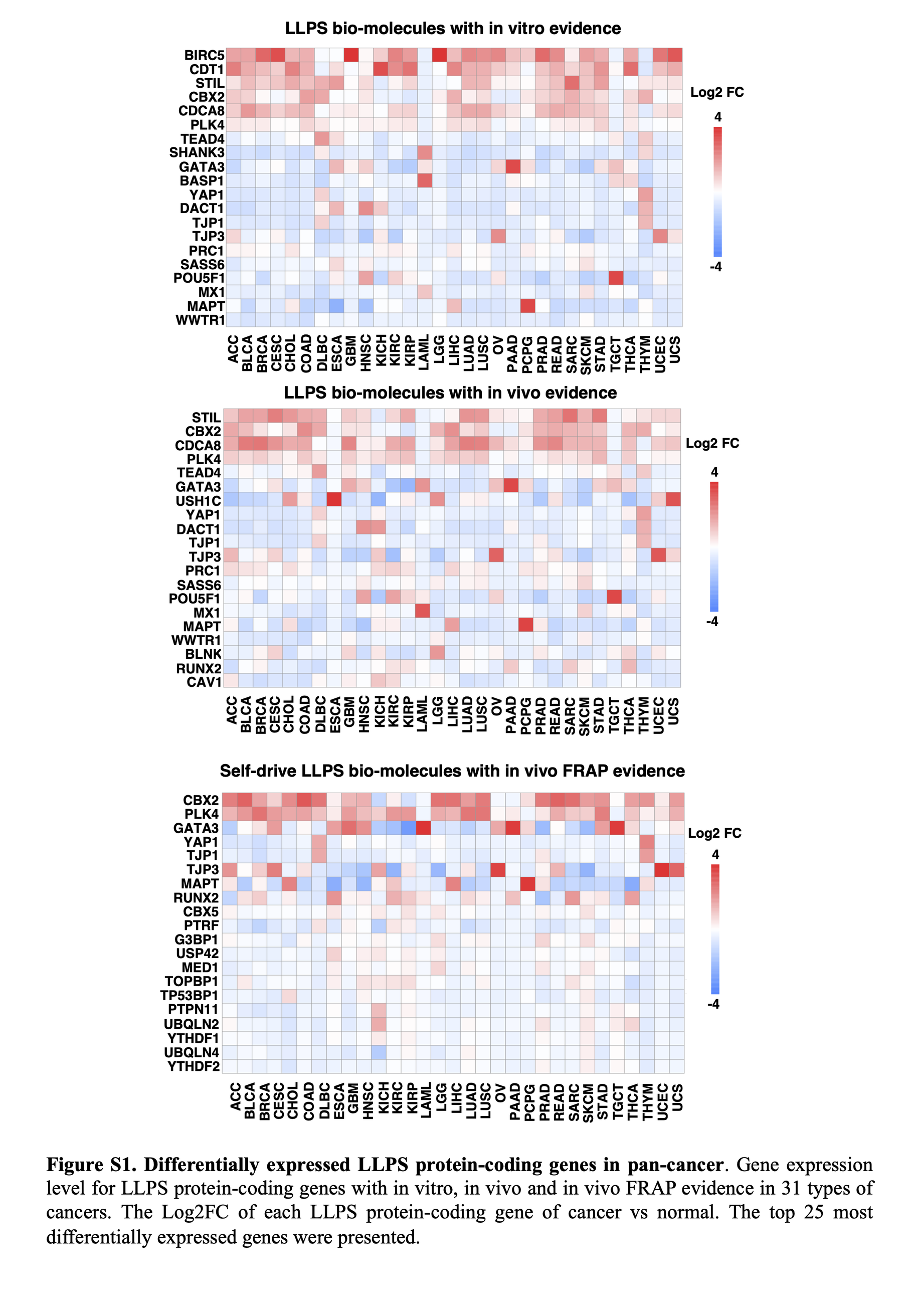

Supplement: S1 Fig — Gene expression level for LLPS protein-coding genes with in vitro, in vivo and in vivo FRAP evidence in 31 types of cancers. The Log2FC of each LLPS protein_coding gene of cancer vs normal. The top 25 most differentially expressed genes were presented. (TIF) [file pone.0287574.s001.tif]

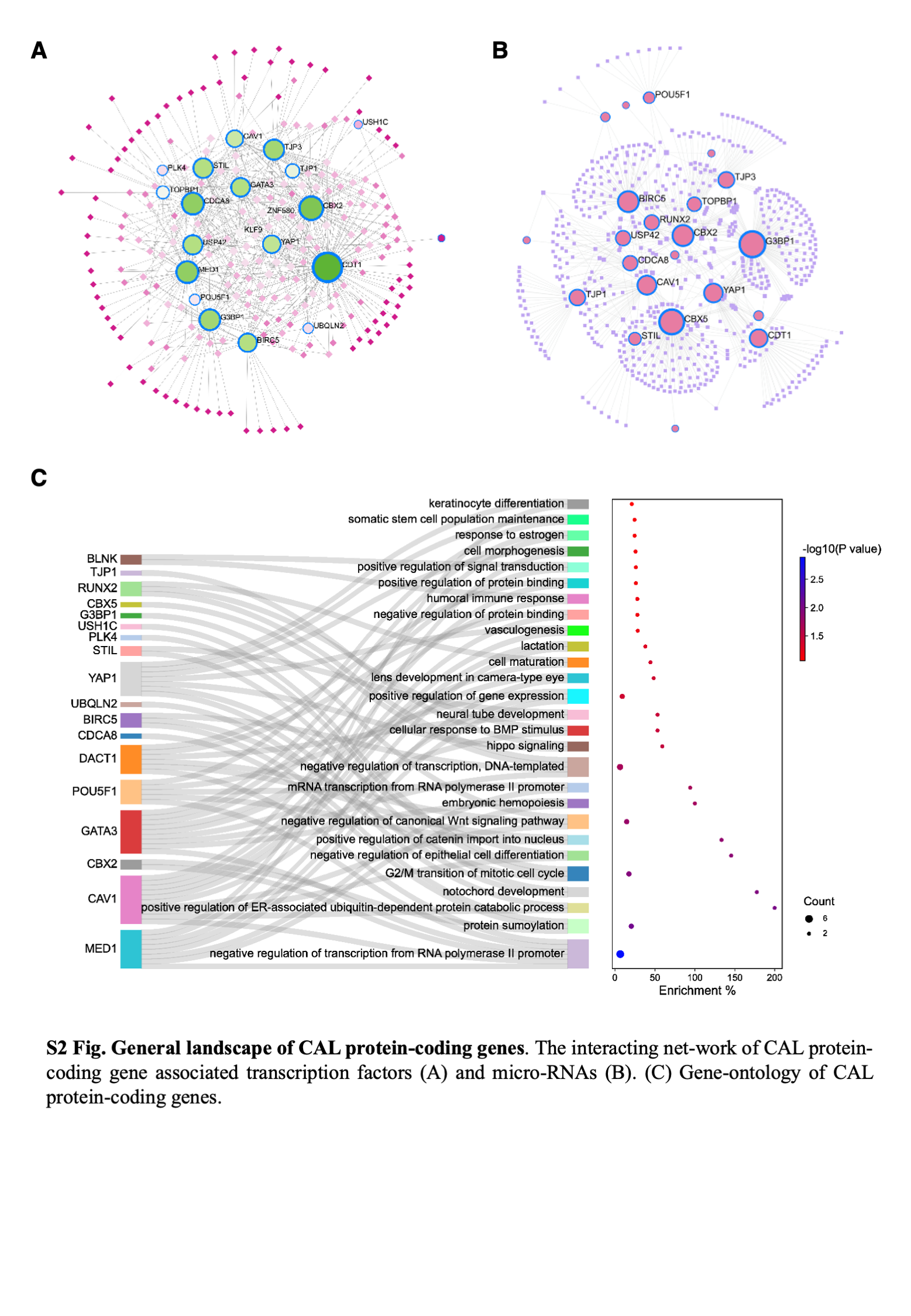

Supplement: S2 Fig — The interaction net-work of CAL protein-coding gene associated transcription factors (A) and micro-RNAs (B). (C) Gene-ontology of CAL protein-coding genes. (TIF) [file pone.0287574.s002.tif]

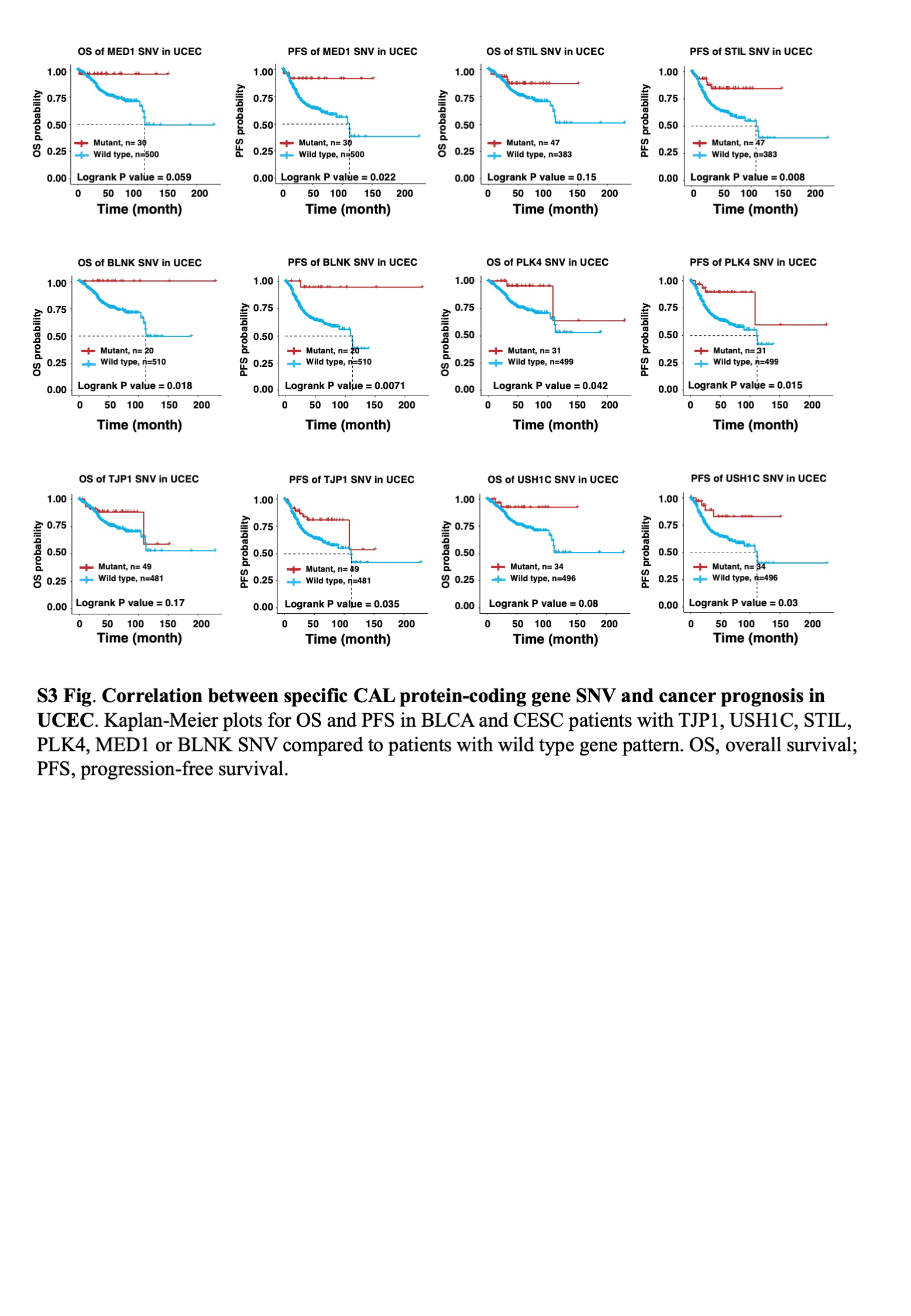

Supplement: S3 Fig — Kaplan-Meier plots for OS and PFS in BLCA and CESC patients with TJP1, USH1C, STIL, PLK4, MED1 or BLNK SNV compared to patients with wild type gene pattern. OS, overall survival; PFS, progression-free survival. (TIF) [file pone.0287574.s003.tif]

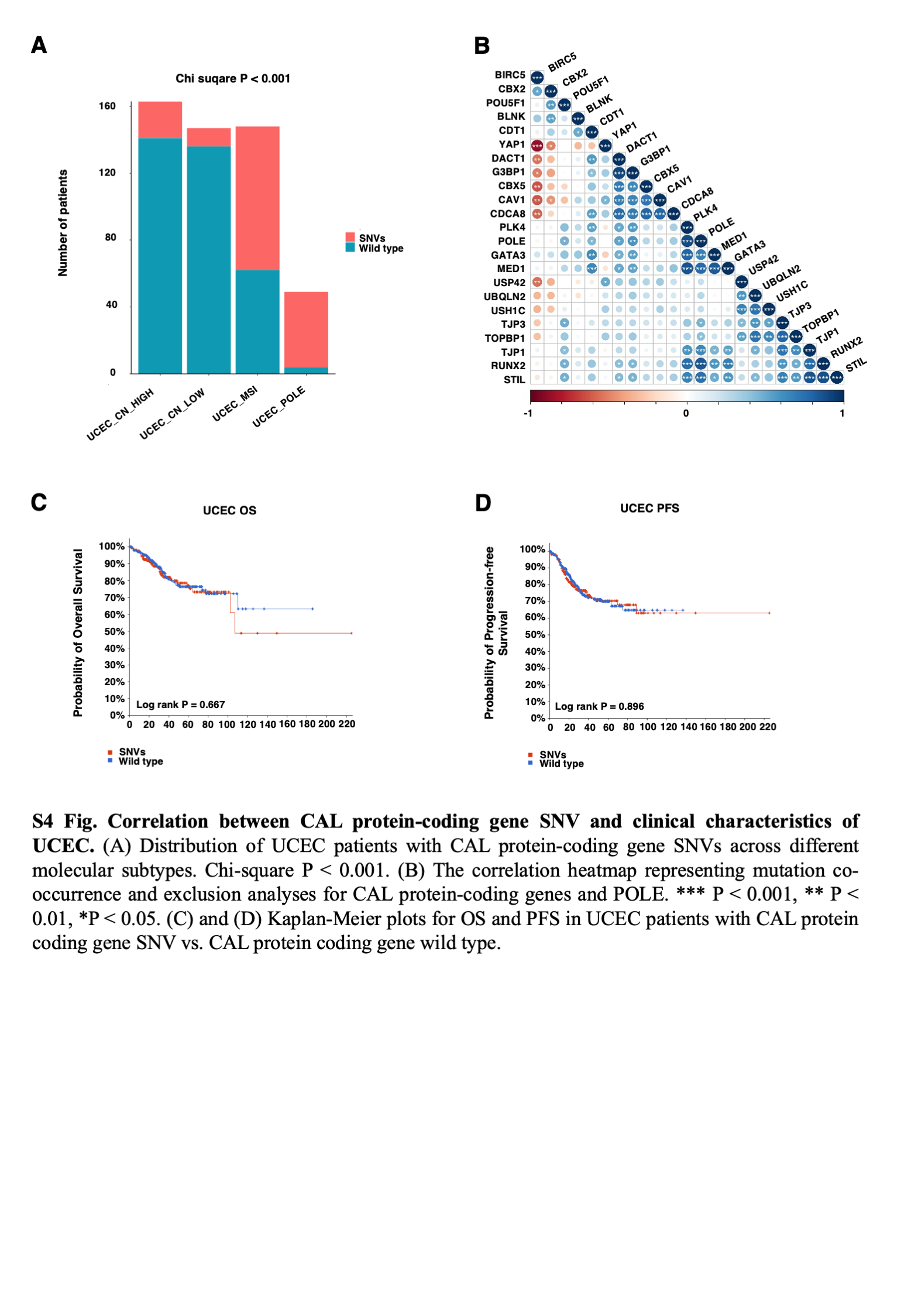

Supplement: S4 Fig — (A) Distribution of UCEC patients with CAL proein-coding gene SNVs across different molecular subtypes. Chi-square P<0.001. (B) The correlation heatmap representing mutation co-occurrence and exclusion analyses for CAL protein-coding genes and POLE. ***p<0.001, **p<0.01, *p<0.05. (C) and (D) Kaplan-Meier plots for OS and PFS in UCEC patients with CAL protein coding gene SNV vs. CAL protein coding gene wild type. (TIF) [file pone.0287574.s004.tif]

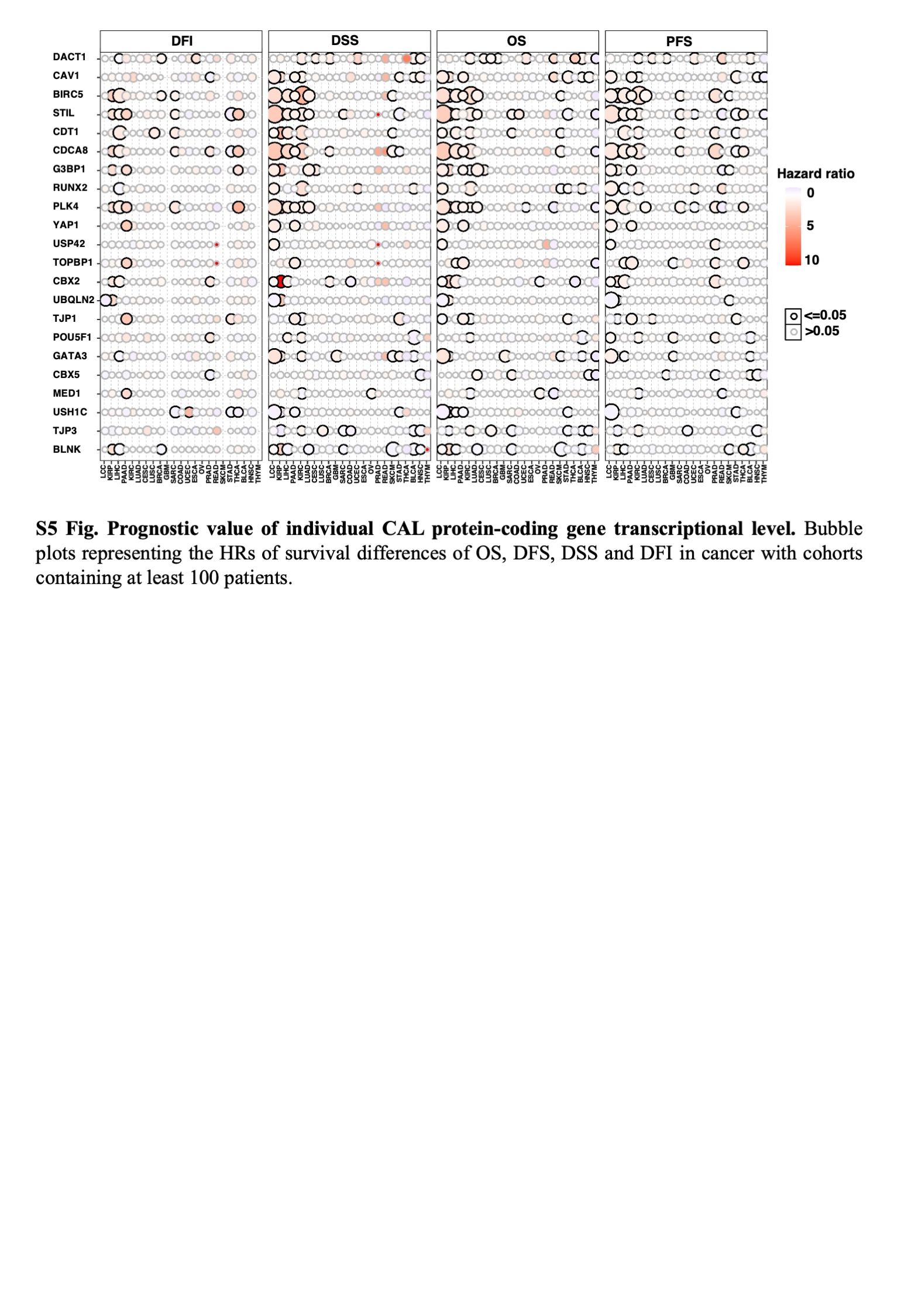

Supplement: S5 Fig — Bubble plots representing the HRs of survival differences of OS, DFS, DSS and DFI in cancer with cohorts containing at least 100 patients. (TIF) [file pone.0287574.s005.tif]

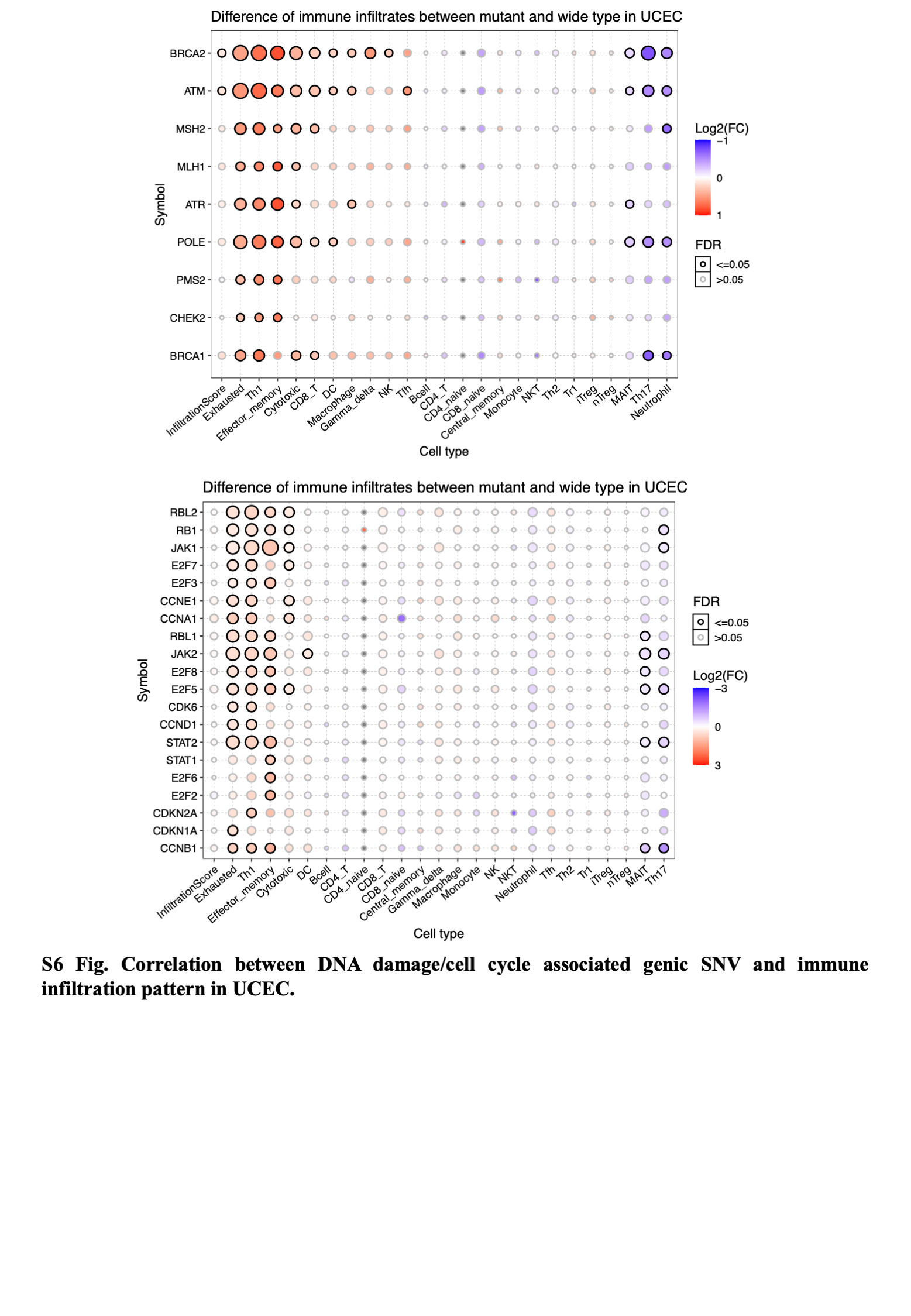

Supplement: S6 Fig — (TIF) [file pone.0287574.s006.tif]

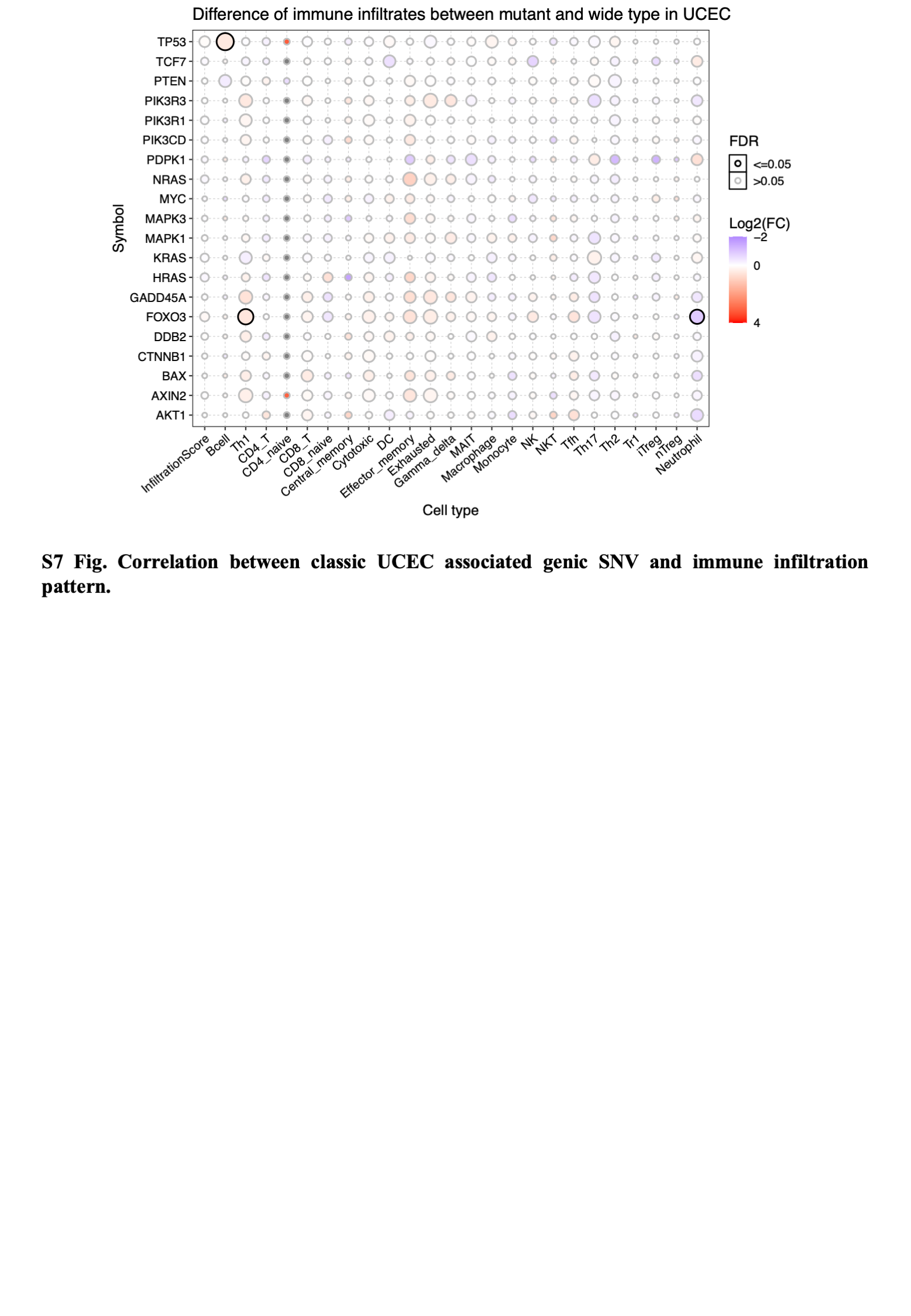

Supplement: S7 Fig — (TIF) [file pone.0287574.s007.tif]

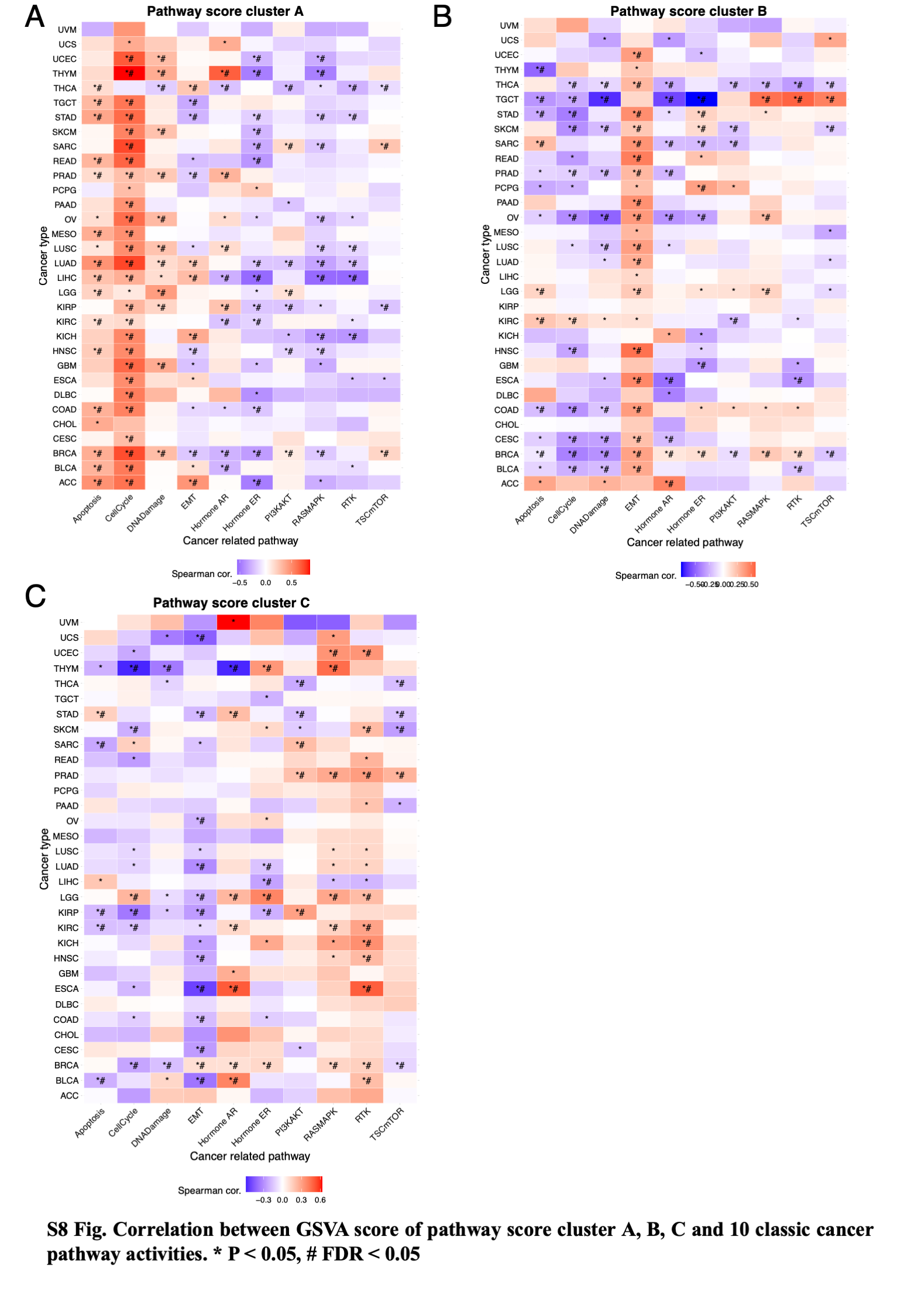

Supplement: S8 Fig — (TIF) [file pone.0287574.s008.tif]

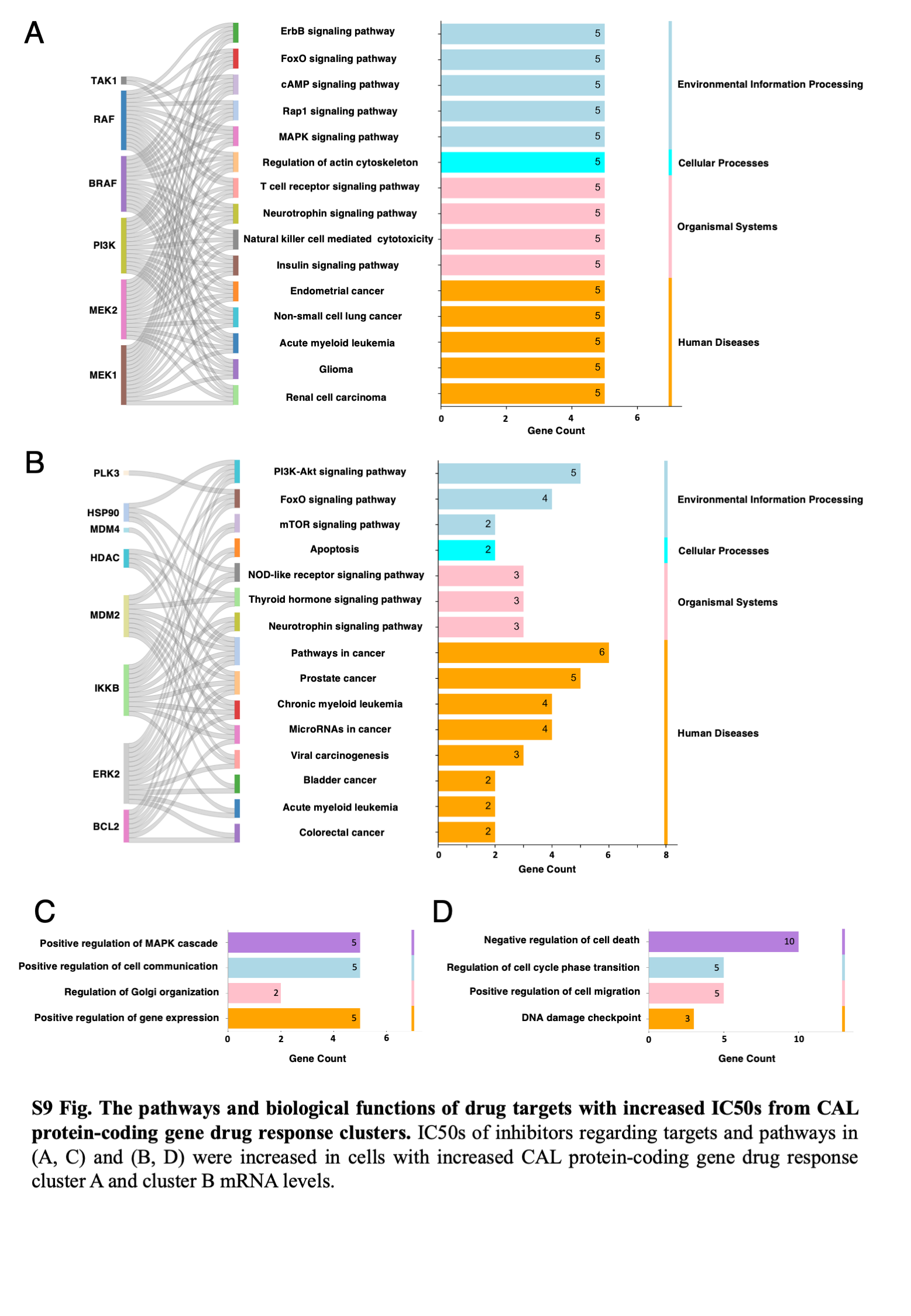

Supplement: S9 Fig — IC50s of inhibitors regarding targets and pathways in (A, C) and (B, D) were increased in cells with increased CAL protein-coding gene drug response cluster A and cluster B mRNA levels. (TIF) [file pone.0287574.s009.tif]

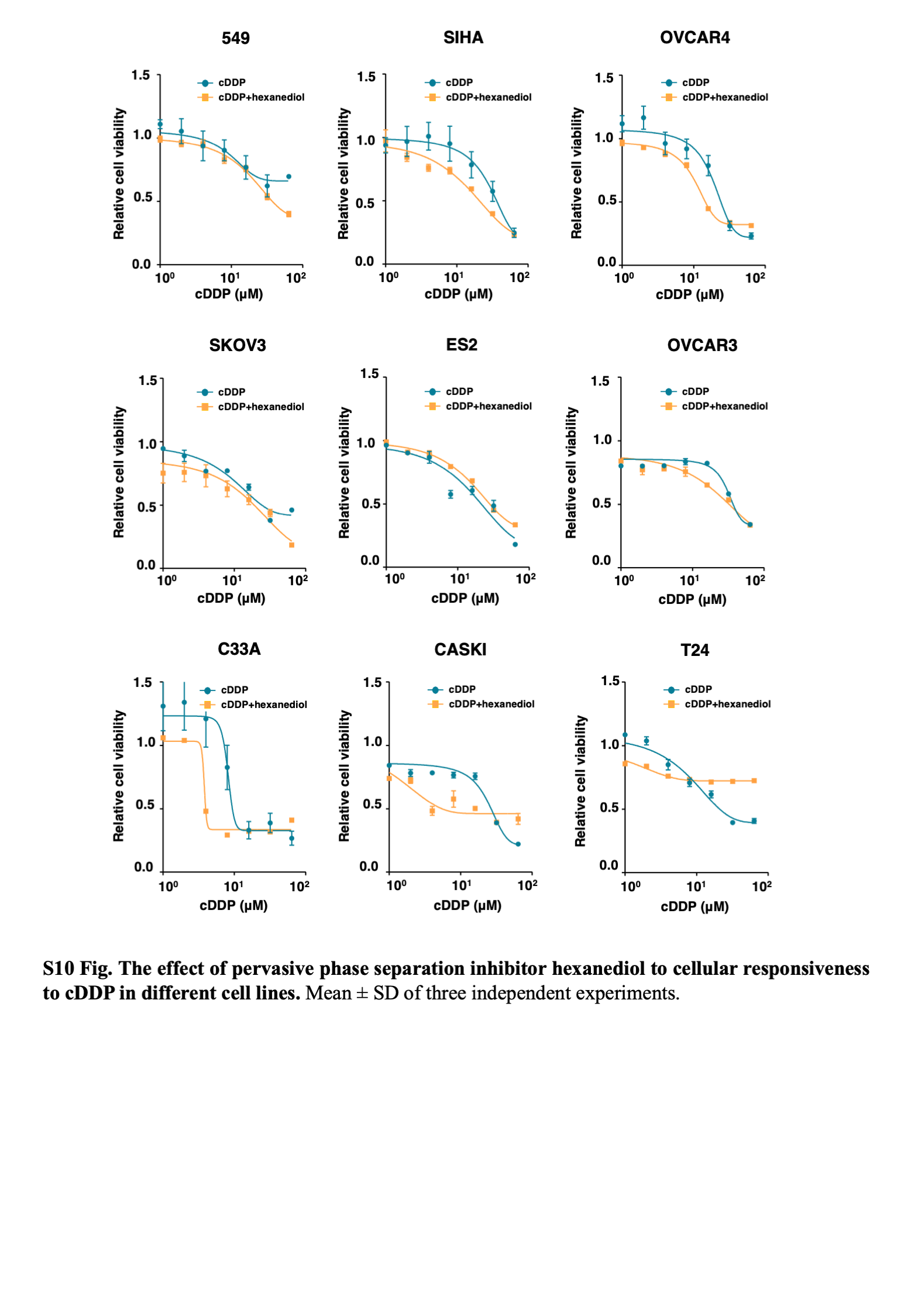

Supplement: S10 Fig — Mean ± SD of three independent experiments. (TIF) [file pone.0287574.s010.tif]

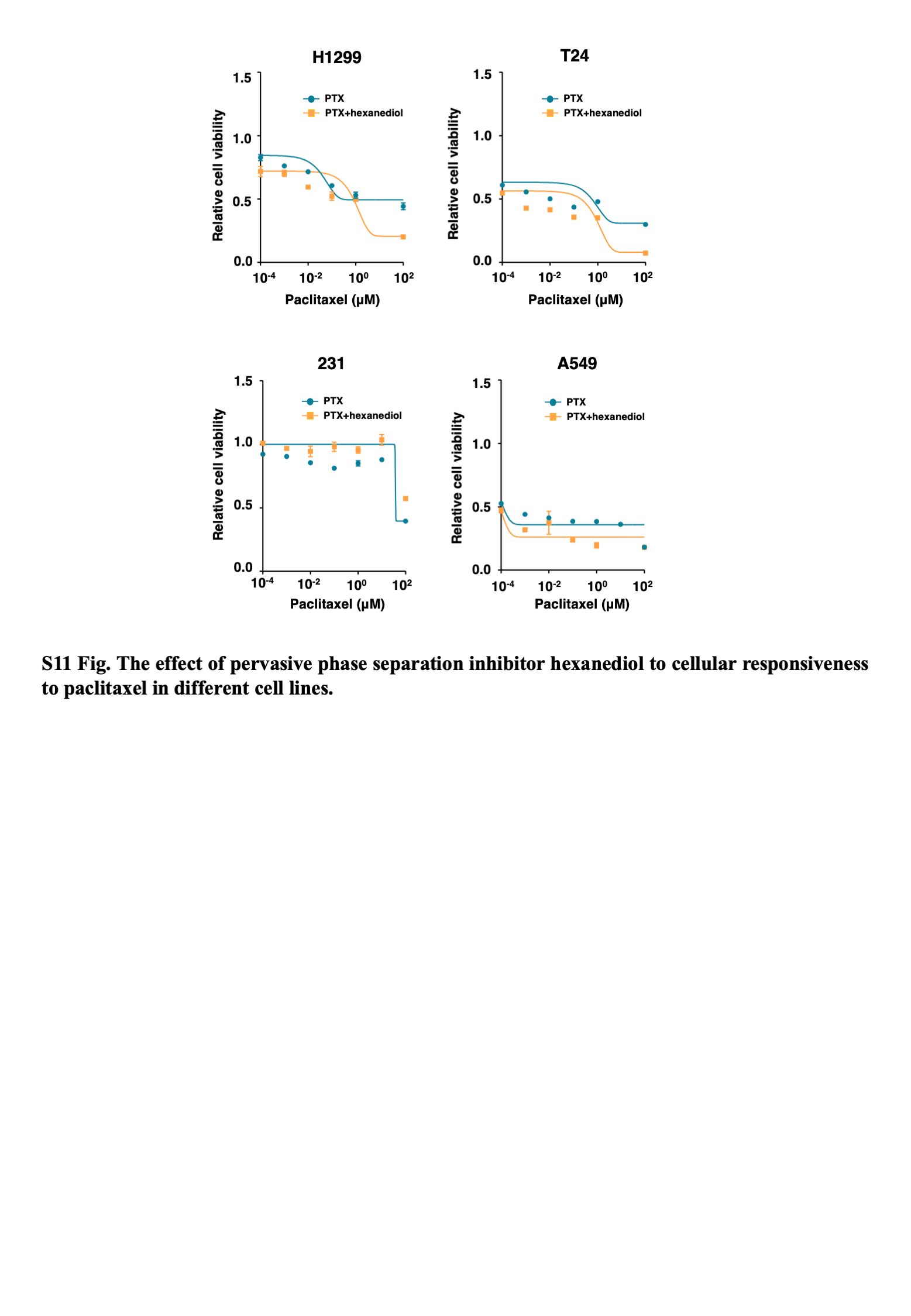

Supplement: S11 Fig — (TIF) [file pone.0287574.s011.tif]

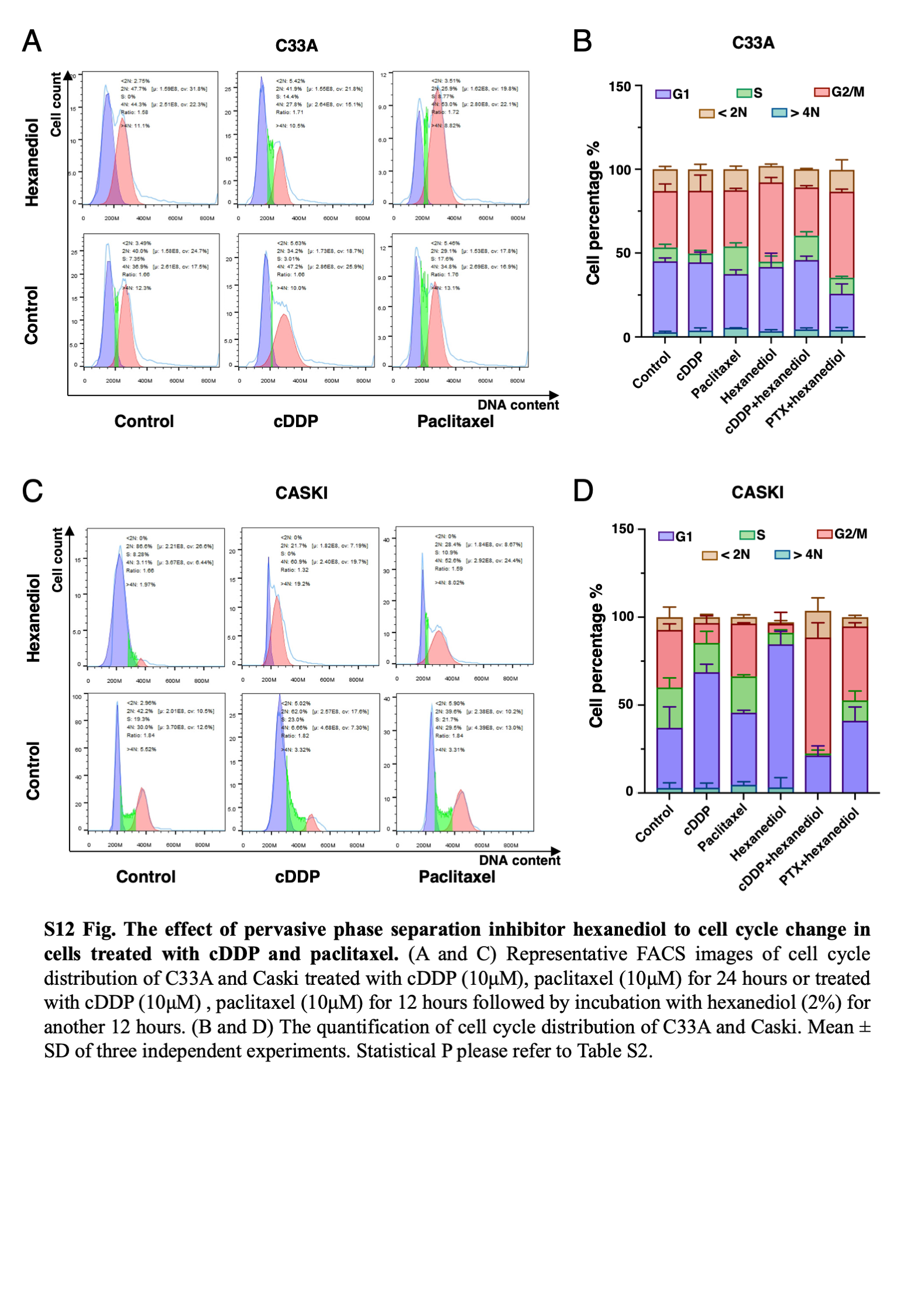

Supplement: S12 Fig — (A and C) Representative FACS images of cell cycle distribution of C33A and Caski treated with cDDP (10μM), paclitaxel (10μM) for 24 hours or treated with cDDP (10μM), paclitaxel (10μM) for 24 hours followed by incubation with hexanediol (2%) for another 12 hours. (B and D) The quantification of cell cycle distribution of C33A and Caski. Mean ± SD of three independent experiments. Statistical P please refer to S2 Table. (TIF) [file pone.0287574.s012.tif]
